# Dual species transcript profiling during the interaction between banana (*Musa acuminata*) and the fungal pathogen *Fusarium oxysporum* f. sp. *cubense*

**DOI:** 10.1186/s12864-019-5902-z

**Published:** 2019-06-24

**Authors:** Wenbin Li, Xiaolin Wang, Chunqiang Li, Jianbo Sun, Shuxia Li, Ming Peng

**Affiliations:** Key Laboratory of Biology and Genetic Resources of Tropical Crops, Institute of Tropical Bioscience and Biotechnology, Chinese Academy of Tropical Agricultural Sciences, Haikou, 571101 Hainan China

**Keywords:** *Musa* spp., *Fusarium oxysporum*, *Fusarium* wilt, Transcriptome, Cytochrome c peroxidase

## Abstract

**Background:**

Banana wilt disease, caused by *Fusarium oxysporum* f. sp. *cubense* Tropical Race 4 (Foc TR4), is one of the most devastating diseases in banana (*Musa* spp.). Foc is a soil borne pathogen that causes rot of the roots or wilt of leaves by colonizing the xylem vessels. The dual RNA sequencing is used to simultaneously assess the transcriptomes of pathogen and host. This method greatly helps to understand the responses of pathogen and host to each other and discover the potential pathogenic mechanism.

**Results:**

Plantlets of two economically important banana cultivars, Foc TR4 less susceptible cultivar NK and susceptible cultivar BX, were used to research the Foc-banana interaction mechanism. Notably, the infected NK had more significantly up-regulated genes on the respiration machinery including TCA cycle, glyoxylate, glycerol, and glycolysis compared to BX at 27 h post inoculation (hpi). In addition, genes involved in plant-pathogen interaction, starch, sucrose, linolenic acid and sphingolipid metabolisms were uniquely more greatly induced in BX than those in NK during the whole infection. Genes related to the biosynthesis and metabolism of SA and JA were greatly induced in the infected NK; while auxin and abscisic acid metabolisms related genes were strongly stimulated in the infected BX at 27 hpi. Furthermore, most of fungal genes were more highly expressed in the roots of BX than in those of NK. The fungal genes related to pathogenicity, pectin and chitin metabolism, reactive oxygen scavenging played the important roles during the infection of Foc. *CCP1* (cytochrome c peroxidase 1) was verified to involve in cellulose utilization, oxidative stress response and pathogenicity of fungus.

**Conclusion:**

The transcriptome indicated that NK had much faster defense response against Foc TR4 than BX and the expression levels of fungal genes were higher in BX than those in NK. The metabolisms of carbon, nitrogen, and signal transduction molecular were differentially involved in pathogen infection in BX and NK. Additionally, the putative virulence associated fungal genes involved in colonization, nutrition acquirement and transport provided more insights into the infection process of Foc TR4 in banana roots.

**Electronic supplementary material:**

The online version of this article (10.1186/s12864-019-5902-z) contains supplementary material, which is available to authorized users.

## Background

Banana (*Musa* spp.) ranks fourth globally in terms of gross value production, after rice, wheat and maize [1]. *Fusarium* wilt in banana, also called Panama disease, is caused by the fungus *Fusarium oxysporum* f. sp. *cubense* (Foc). The most virulent race of Foc for ‘Cavendish’ banana is Foc tropical race 4 (Foc TR4). This fungus severely limits the productivity of the leading Cavendish banana cultivar, resulting in drastic economic losses throughout the banana-producing regions [2]. The vascular wilt fungus *Fusarium oxysporum* is an asexual, soil inhabiting facultative parasite [3]. *Fusarium oxysporum* has different asexual spores: microconidia directly penetrate the living roots, macroconidia usually colonize the surface of dead plants, and chlamydospores lie dormant in soil for decades [4, 5]. Foc causes severe diseases in many economically important crops, including tomato (*Solanum lycopersicum* L.) [6], cucumber (*Cucumis sativus* L.) [7], and cotton (*Gossypium hirsutum* L.) [8]. A seedling plant infected by Foc shows drastic physiological and morphological alterations, including slowed growth, leaf chlorosis, and finally, whole plant wilt. The fungal colonization disrupts the transportation of nutrition and water of plants through vascular tissue. In response to this biotic stress, plants induce a range of immune responses, including physical barriers (e.g., cell wall-associated compounds) [9] and molecular responses (e.g., hypersensitive response, production of reactive oxygen species, activation of signal molecules, and expression of pathogen-related genes) [10].

High-throughput sequencing technology, especially the RNA-seq, facilitates the more precise molecular changes of plants that accompany biotic and abiotic stress. In previous researches, many banana plant genes related to defense-related pathways mainly including phenylpropanoid biosynthesis, phenylalanine metabolism, carbon metabolisms, amino acid recycling, hormone signal transduction and plant-pathogen interaction were greatly induced in the face of Foc TR4 [11–14]. Meanwhile, many fungal genes related to virulence, transporters and transcription factors for toxin and nutrient, signaling pathways, and adaptation to host were strongly activated in the co-culture with banana roots [15]. Recently, the dual RNA-seq has become a powerful method to comprehensively understand the interaction between host and pathogen in vivo [16]. This dual sequencing uncovers biosynthetic and metabolic pathways of cross-talk from participants simultaneously and specifically links the dynamic expression profiles of genes with the interaction [17, 18]. However, the mutual response of attack and counterattack in vivo between banana and fungus has been poorly known till now.

In the present work, the susceptible cultivar ‘Baxi’ (BX) and its less susceptible mutant cultivar ‘Nongke No. 1’ (NK) to Foc TR4 were employed for Foc infection [12, 19]. NK is bred from BX clones and has the similar growth period and morphology of fruits with BX; however, it has the higher soluble sugar and less susceptible to Foc TR4 than BX [20]. To research the infection response at the early stage, the banana sampling times of 27 and 51 h post inoculation (hpi) were determined according to our previous research [13].

This study begins with a comprehensive transcriptomic analysis of the infected banana roots and Foc TR4 in vivo using the dual RNA-seq method. The differentially expressed banana genes from the infected BX and NK indicated the potential response mechanisms of different banana cultivars to Foc. Moreover, the expression levels of fungal genes in different hosts at two time points were investigated at an unprecedented depth. The faster response of banana defense genes and the lower fold change of fungal genes in NK compared to BX might determine the less susceptibility of NK. Furthermore, fungal gene encoding cytochrome c peroxidase (CCP1) was investigated to play a role in cellulose utilization. The low disease incidence in BX plantlets infected by the *CCP1* mutant strains indicated that *CCP1* might play an important role in fungal pathogenicity.

Overall, this transcriptome dataset allows us to gain insights into the complex banana-Foc TR4 interaction and to investigate the potential pathogenesis of Foc. Moreover, it would benefit to attenuate/control fungal pathogenicity or breed the resistant cultivars.

## Results

### Symptoms of the infected banana plants

**Fig. 1:**
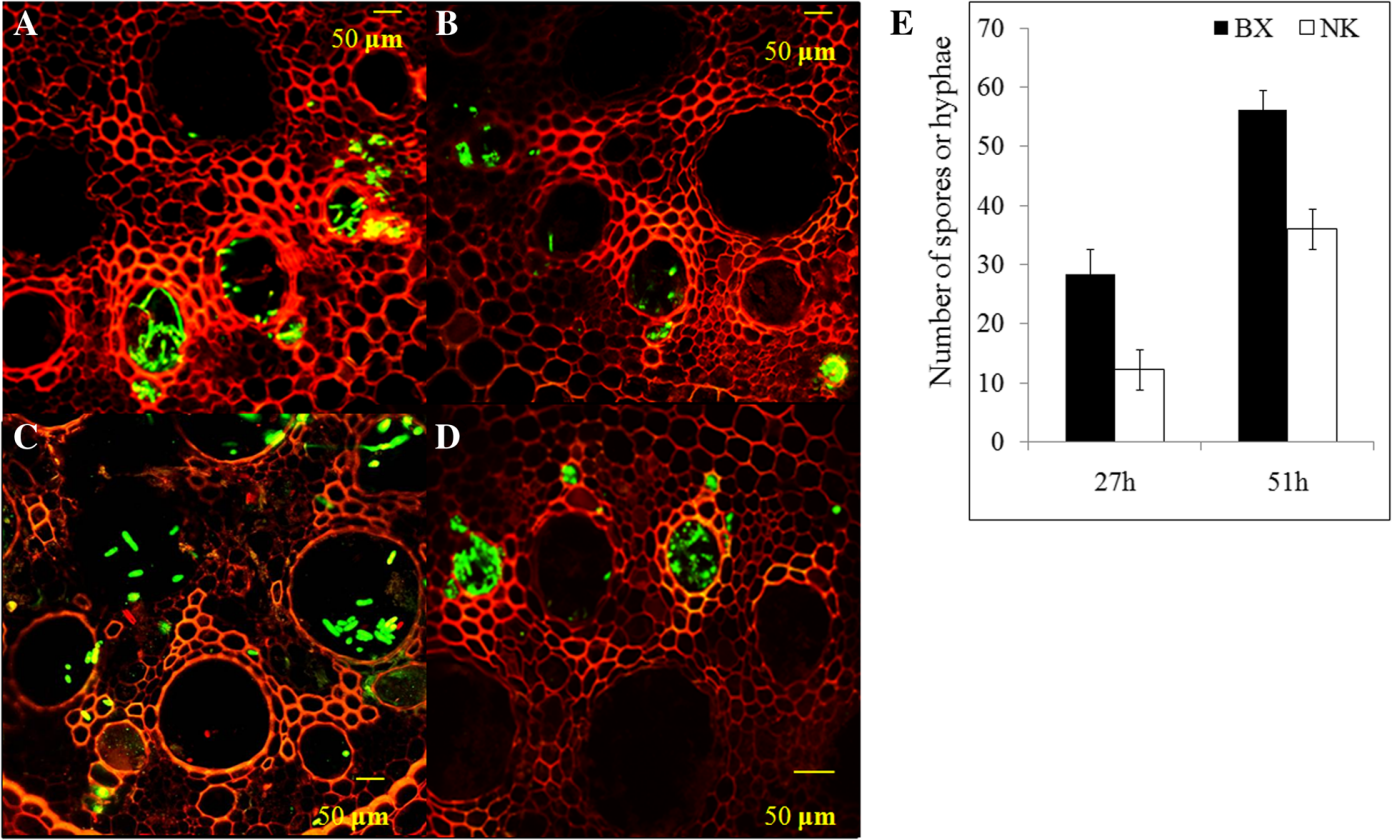
The infection process and the number of conidia and hyphae in banana roots at the early infection stage. At 27 h post inoculation (hpi), conidia and hyphae of the GFP-expressing Foc TR4 were observed in the vascular tissue of BX (**a**) and NK (**b**) banana. At 51 hpi, more conidia and hyphae were found in BX (**c**) and NK (**d**) compared to the fungal presence at 27 hpi (**e**) The number of conidia or hyphae in roots under laser confocal microscope. BX: the infected BX roots, NK: the infected NK roots. A total of 60–80 root transverse sections were counted for every treatment samples for three replications. The error bars represent SE. Asterisks indicate significantly difference (* *P* < 0.05) through Student’s test.

The banana roots inoculated with GFP-tagged Foc TR4 were used to inspect the development of fungal colonization. The GFP strains of Foc TR4 had no difference on morphology and pathogenesis with their wild strains [21]. Hyphae and conidia were found throughout the vascular bundles radiating from the infection point. A greater number of hyphae and conidia were found at 51 hpi compared with at 27 hpi in both infected-banana cultivars (Fig. 1). In addition, the number of hyphae and conidia in BX roots (Fig. 1a and c) was significantly higher than that in NK (Fig. 1b and d) at both time points (Fig. 1e), indicating that Foc might infected into BX roots more easily and quickly than NK roots. The number of hyphae means the higher efficiency of directly touched-infection in our research compared to those with dipped-roots infection in previous researches, which might strengthen the response between host and fungus. At 3 hpi, the amount of hyphae and conidia in NK roots were not enough for gathering fungal material for RNA-Seq. At about 45 days after infection, BX plants showed the visible wilt disease symptom, i.e. the yellow leaves and the rotten blotches in the vascular of the rhizome, while NK leaves kept green and roots were still healthy (Additional file 1: Figure S1).

### Banana root transcriptomes during the early stages of Foc TR4 infection

The RNA-seq transcriptome data from our previous report [22], which focused on identification of long non-coding RNAs, were more widely analyzed here. Briefly, RNA was extracted from NK and BX roots inoculated with Foc TR4 at two time points (27 and 51 hpi) from three biological replicates. Of the 36,542 banana genes predicted within the genome, 45,832 transcripts were mapped to the banana transcriptome data under this condition. In each pair-wise comparison using Cuffdiff, more differentially expressed banana genes (DEGs) were induced in the infected NK than in the infected BX at 27 hpi (2322 vs. 1887) compared to their mock-inoculated samples; however, fewer DEGs were found in mock-inoculated NK than those in mock-inoculated BX at the same time (1466 vs. 2565). It means that NK responds more quickly to Foc TR4 at the early stage than BX (Table 1).

**Table 1:** Number of differentially expressed genes (DEGs) in susceptible and less susceptible banana cultivars during *Foc* infection

Time	Log_2_^(BX/BX-MK)^		Log_2_^(NK/NK-MK)^		Log_2_^(NK/BX)^		Log_2_^(NK-MK/BX-MK)^	
	Up	Down	Up	Down	Up	Down	Up	Down
27 hpi	1098	789	1373	949	195	549	457	1009
51 hpi	1287	619	977	1095	164	691	811	1754

hpi: hours post inoculation, *q-value* < 0.05, |Log_2_^Fold change^| ≥1, MK: mock-inoculated samples

**Fig. 2:**
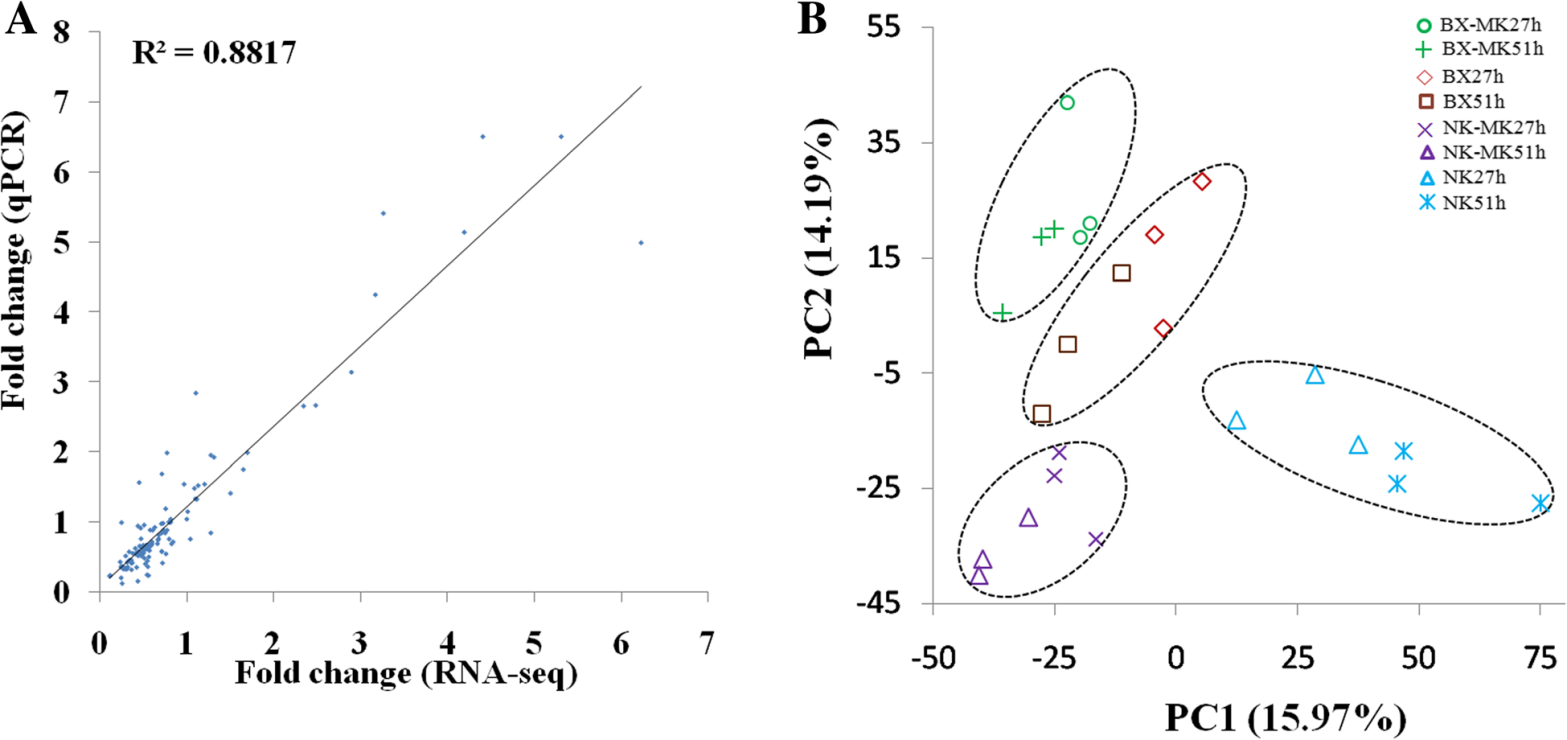
qPCR evaluation of RNA-seq data from Foc-infected banana cultivars. **a** Comparison of gene expression values obtained by qPCR and RNA-seq. Fold-change values were calculated for 30 banana genes from the infected sample comparing to the mock-inoculation sample and 10 *Foc* genes from the infected sample comparing to from the infected BX at 27 h. **b** Principal Components Analysis (PCA) displaying the intrinsic biological variation among banana samples. The result confirms the clear distinction between the transcriptomes of infected and healthy plants. Circle, mock-inoculated BX at 27hpi (BX-MK27h); green cross, mock-inoculated BX at 51 hpi (BX-MK51h); diamond, infected BX at 27 hpi (BX27h); square, infected BX at 51 hpi (BX51h); purple cross, mock-inoculated NK at 27 hpi (NK-MK27h); purple triangle, mock-inoculated NK at 51 hpi (NK-MK51h); blue triangle, infected NK at 27 hpi (NK27h); and asterisk, infected NK at 51 hpi (NK51h)

The expression levels of 30 banana genes and 10 fungal genes from RNA-seq were validated by qPCR assays (Additional file 2: Table S1). We observed a strong correlation (R^2^ = 0.88) between the results obtained using the two techniques (Fig. 2a). Principal component analysis (PCA) was performed on the biological variability across all samples. The result revealed that four groups were distinctly formed; moreover, a greater overall change was found in NK than in BX (Fig. 2b).

### Banana roots showed up-regulation of transcripts related to energy metabolism during infection

**Fig. 3:**
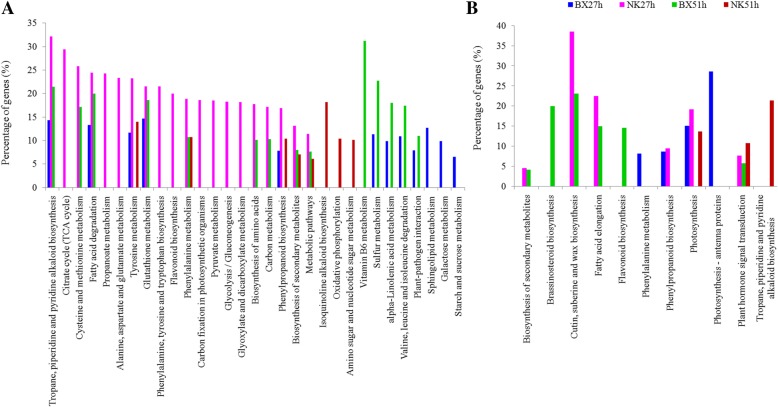
Metabolic pathways identified through KEGG analysis on the DEGs in two banana cultivars. The DEGs were obtained with the threshold of |Log_2_^(inoculated/mock-inoculated)^| ≥ 1 and *q* value < 0.05. Blue, BX vs. mock-inoculated BX at 27 hpi; Pink, NK vs. mock-inoculated NK at 27 hpi; Green, BX vs. mock-inoculated BX at 51 hpi; Red: NK vs. mock-inoculated NK at 51 hpi. **a** The up-regulated DEGs. **b** The down-regulated DEGs. The percentage of gene means the ratio of input number to the background number

The DEGs were compared to the Kyoto Encyclopedia of Genes and Genomes (KEGG) and Gene Ontology (GO) databases under the control of q value < 0.05. More up-regulated DEGs were exclusively found in the infected NK at 27 hpi than in the infected BX (Fig. 3a). Furthermore, twenty pathways from the up-regulated genes were significantly enriched in NK at 27 hpi especially citrate cycle and amino acids metabolism and biosynthesis. Seven pathways were uniquely enriched in BX at 27 hpi, except for five pathways shared with NK. At 51 hpi, 8 and 11 pathways were significantly enriched in NK and BX, respectively, of which 3 pathways were shared in both cultivars. Other DEGs related to PAMP, pathogenesis-related proteins (PRs), oxidative burst and cell wall modification were highly induced in the infected NK at 27 hpi (Additional file 3: Table S2). A repression of the DEGs related to cutin, suberine and wax biosynthesis and fatty acid elongation were greatly clustered in NK at 27 hpi and in BX at 51 hpi (Fig. 3b). In addition, those DEGs related to phenylalanine metabolism and photosynthesis were only greatly depressed in the infected BX at 27 hpi.

The GO term analysis also uncovered the differences between the infected BX and NK under q value < 0.05. For instance, the DEGs with organic substance metabolic process and molecular functions were uniquely induced in NK, as eleven GO terms were not distributed by NK at either time point. In addition, the down-regulated DEGs from two cultivars distributed in different terms except for ‘heme and tetrapyrole binding’ (Additional file 4: Figure S2).

We also investigated the function enrichment of the DEGs between mock-inoculated BX and NK cultivars (DEMG) through KEGG and GO. The up-regulated DEMGs were mainly related to citrate cycle and phenylalamine metabolism, cutin, suberine, wax, steroid biosynthesis through KEGG analysis (Additional file 5: Figure S3). The down-regulated DEMGs were mainly clustered in cysteine, methionmine, glycerolipid metabolism and fatty acid elongation. Under GO analysis, the up-regulated DEMGs were only clustered at 27 hpi, and they greatly distributed in biological, metabolic processes, and catalytic activity terms. The enriched up-regulated DEMGs at 51 hpi uniquely distributed into the terms of molecular function except for the cofactor binding (Additional file 6: Figure S4). DEGs from the infected banana roots, related to ‘plant-pathogen interaction’, ‘hormone signal transduction’ or ‘phenylpropanoid biosynthesis’, were not found in these DEMGs.

### Insight into signal molecules metabolism in infected banana plants

**Fig. 4:**
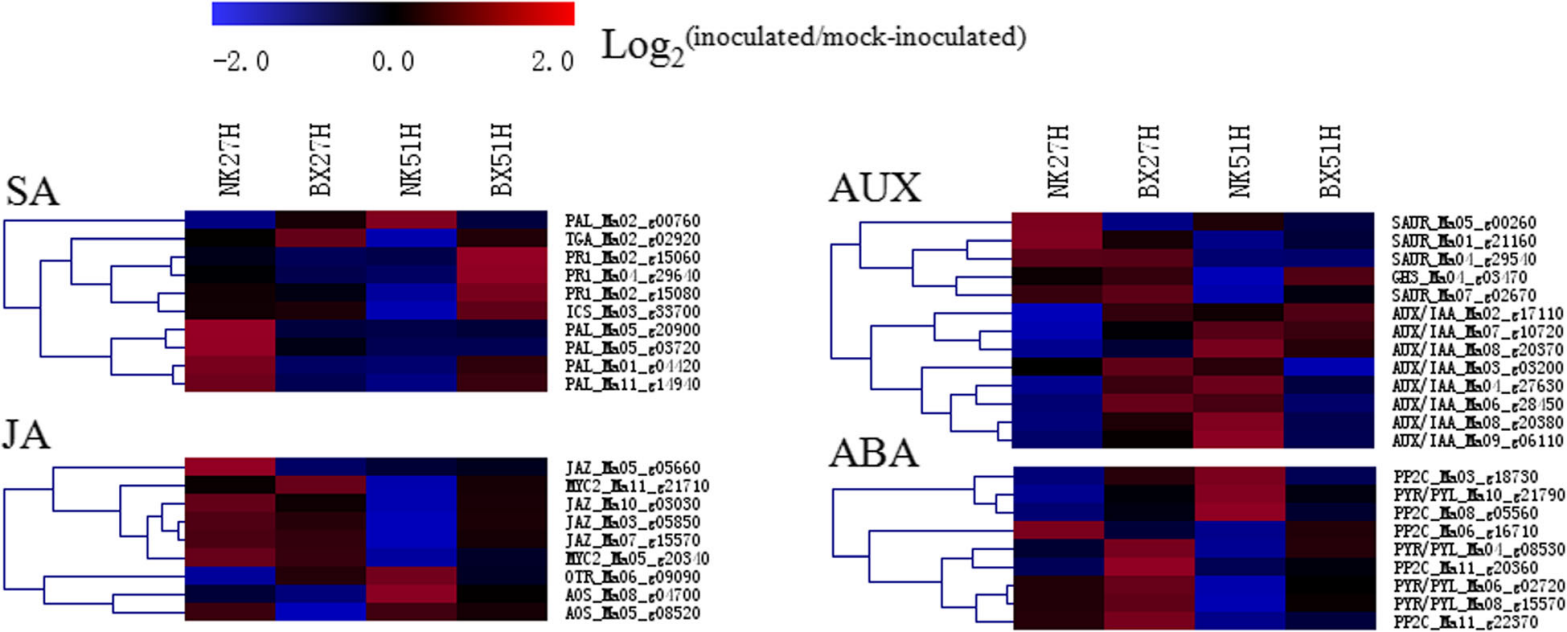
The expression profiles of the DEGs related to SA, JA, auxin and abscisic acid (ABA) signal transduction in two banana cultivars. Each row represents one candidate gene. The expression fold change of the infected over mock-inoculated sample at the same time point is represented by a color scale ranging from saturated blue (− 2) to saturated red (2)

The signal molecules salicylic acid (SA), jasmonic acid (JA), auxin, and abscisic acid (ABA) extensively respond to the biotic and abiotic stresses in plant. In our research, many DEGs related to the biosynthesis and metabolism of SA, JA, auxin, and ABA were also found in banana roots (Fig. 4), and their expression levels were verified through qRT-PCR (Additional file 7: Table S3).

#### Salicylic acid and Jasmonic acid

More DEGs involved in SA biosynthesis and metabolism were greatly up-regulated in NK compared to those in BX at 27 hpi, including phenylalanine ammonia-lyase (*PAL*) and pathogenesis-related protein 1 (*PR-1*) genes (Fig. 4). By contrast, more DEGs were greatly up-regulated in BX than those in NK at 51 hpi. As for JA, all jasmonate ZIM domain-containing protein (*JAZ*) genes, a negative regulator in JA transduction, had the higher expression changes in NK than those in BX at 27 hpi. The 12-oxophytodienoate reductase (*OTR*) and allene oxide synthase (*AOS*) genes, responsible for the biosynthesis of JA, had higher expression fold changes at 51 hpi compared to those at 27 hpi in both cultivars. These genes expression profiles were consistent with our previous data: the infected NK had a higher concentration of SA and a lower concentration of JA than the infected BX at 27 hpi [23].

#### Auxin

Primary auxin-responsive DEGs fall into three major classes: auxin responsive GH3 gene family (GH3), auxin/indole-3-acetic acid protein (AUX/IAA), and small auxin-up RNA (SAUR). Most of three families genes were greatly induced in the infected BX at 27 hpi, and half of them were depressed at 51 hpi. In our previous research, the level of 3-indoleacetic acid was lower in BX roots at 51 hpi than that at 27 hpi [23]. The depressed *AUX*/*IAA* genes might indicate the lower level of 3-indoleacetic acid in NK at 27 hpi than that in BX.

#### Abscisic acid

The DEGs encoding abscisic acid receptor (PYR/PYL) and protein phosphatase 2C (PP2C) were more induced in BX compared to in NK at 27 hpi. At 51 hpi, more than half of these genes were depressed in NK, suggesting that the metabolism of ABA might be inhibited in NK (Fig. 4). The higher fold changes of these DEGs at 27 hpi than those at 51 hpi also verified the peak level of ABA at 27 hpi in BX [23].

Some DEGs (mean FPKM > 10) related to cytokinin (A-ARR family), gibberellin (DELLA protein), brassinosteroid (shaggy-related protein kinase eta-like isoform X1, BIN2), and ethylene (ethylene-responsive transcription factor 1, ERF1/2) biosynthesis and metabolism were also found in both banana cultivars (Additional file 8: Table S4).

### Identification and functional analysis of fungal transcripts present in banana roots

The RNA-seq data was also mapped to the Foc TR4 genome with 18,065 potential protein coding genes. About 8% of the reads from the infected samples (14,459 transcripts) were mapped to the Foc TR4 genome (Additional file 9: Table S5). When the data were tallied under a less stringent threshold (> 0.5 FPKM), 10,777 fungal transcripts accounting for about 60% of the predicted fungal genes were obtained in this research. It was notable that there were more fungal genome mapped-reads present in BX roots than in NK (6.29% vs. 3.23% at 27 hpi and 2.23% vs. 1.59% at 51 hpi). Additionally, more up-regulated differentially expressed fungal genes (DEFGs) were from BX roots compared to those from NK roots at both time points (Additional file 10: Table S6).

**Fig. 5:**
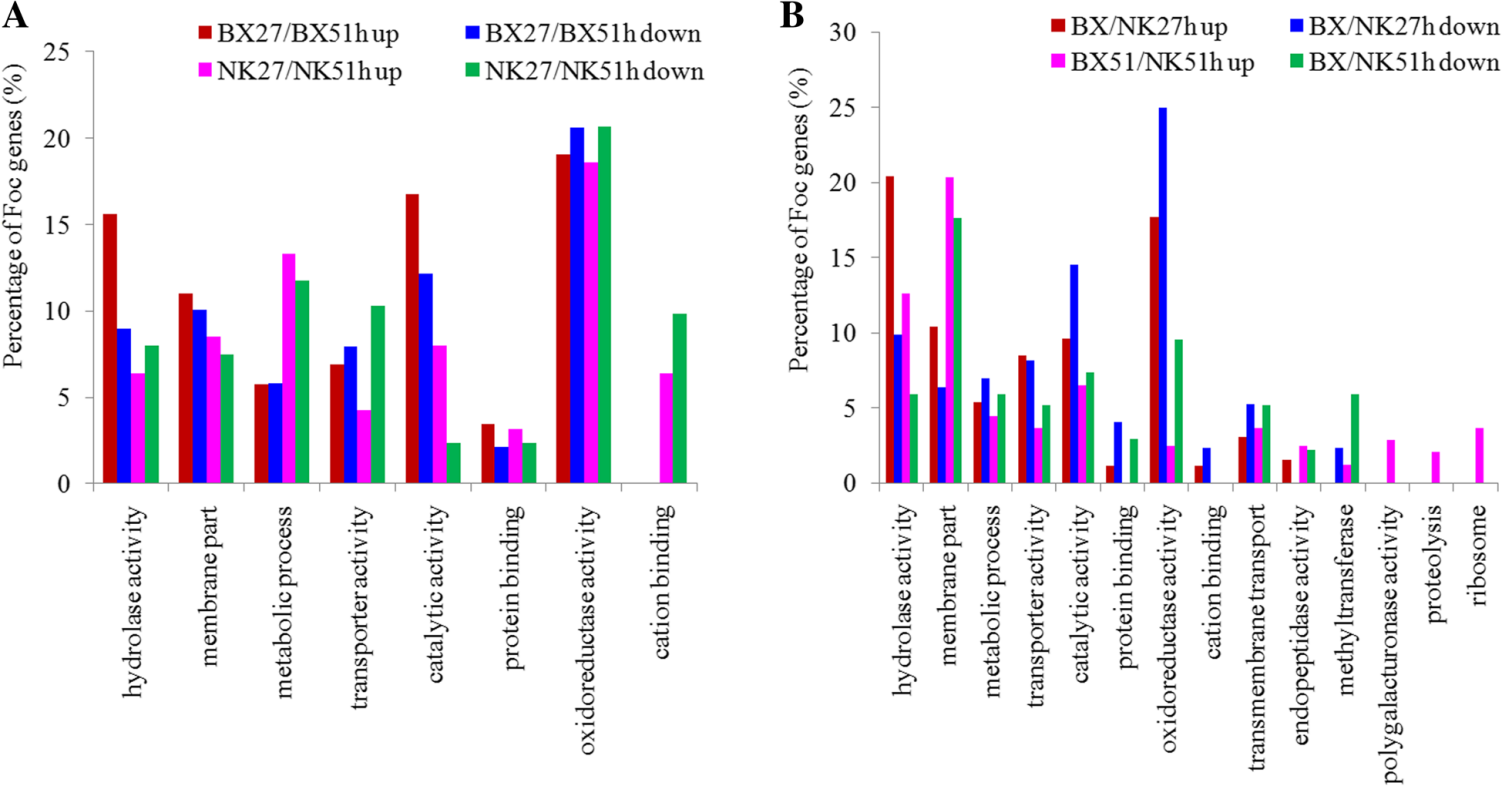
Metabolic GO pathways of differentially expressed fungal genes (DEFGs) present in two banana cultivars. Bar graph of GO classification of fungal genes differentially expressed in pair-wise comparisons. **a** Comparisons between two time points: Red, the up-regulated genes in BX at 27 hpi compared to at 51 hpi; Pink, the up-regulated genes in NK at 27 hpi compared to at 51 hpi; Blue, the down-regulated genes in BX at 27 hpi compared to at 51 hpi; Green, the down-regulated genes in NK at 27 hpi compared to at 51 hpi. **b** Comparisons between two cultivars: Red, the up-regulated genes in BX compared to NK at 27 hpi; Pink, the up-regulated genes in BX compared to NK at 51 hpi; Blue, the down-regulated genes in BX compared to NK at 27 hpi; Green, the down-regulated genes in BX compared to NK at 51 hpi. The percentage of genes means the ratio of DEFGs number in this bin to the total DEFGs number and the total number of DEFGs was shown in Additional file 10: Table S6

GO-based enrichment analyses were performed on the DEFGs. The DEFGs from different time points were mainly enriched in eight categories (Fig. 5a, comparing 27 and 51 hpi). The percentages of the up-regulated DEFGs in most of categories were higher in BX (red column) than those in NK (purple column), except for the terms of metabolic process and cation binding. By contrast, the percentages of the down-regulated DEFGs related to metabolic process, transporter activity and cation binding were higher in NK (green column) than those in BX (blue column). By comparing the DEFGs at the same time between two cultivars, other trends could be seen (Fig. 5b, comparing BX and NK). At 27 hpi, the up-regulated DEFGs were mainly involved in hydrolase and member activities (red column), while the down-regulated DEFGs mainly distributed in oxidoreductase and catalytic activity (blue column). Remarkably, the percentages of the DEFGs involved in membrane part were the highest at 51 hpi (purple and green columns). In addition, some up-regulated DEFGs at 51 hpi uniquely distributed in polygalacturonase, proteolysis and ribosome activities. Fewer DEFGs were enriched with KEGG analysis (data not shown).

### Characterizing the fungal genes expressed within plants

**Fig. 6:**
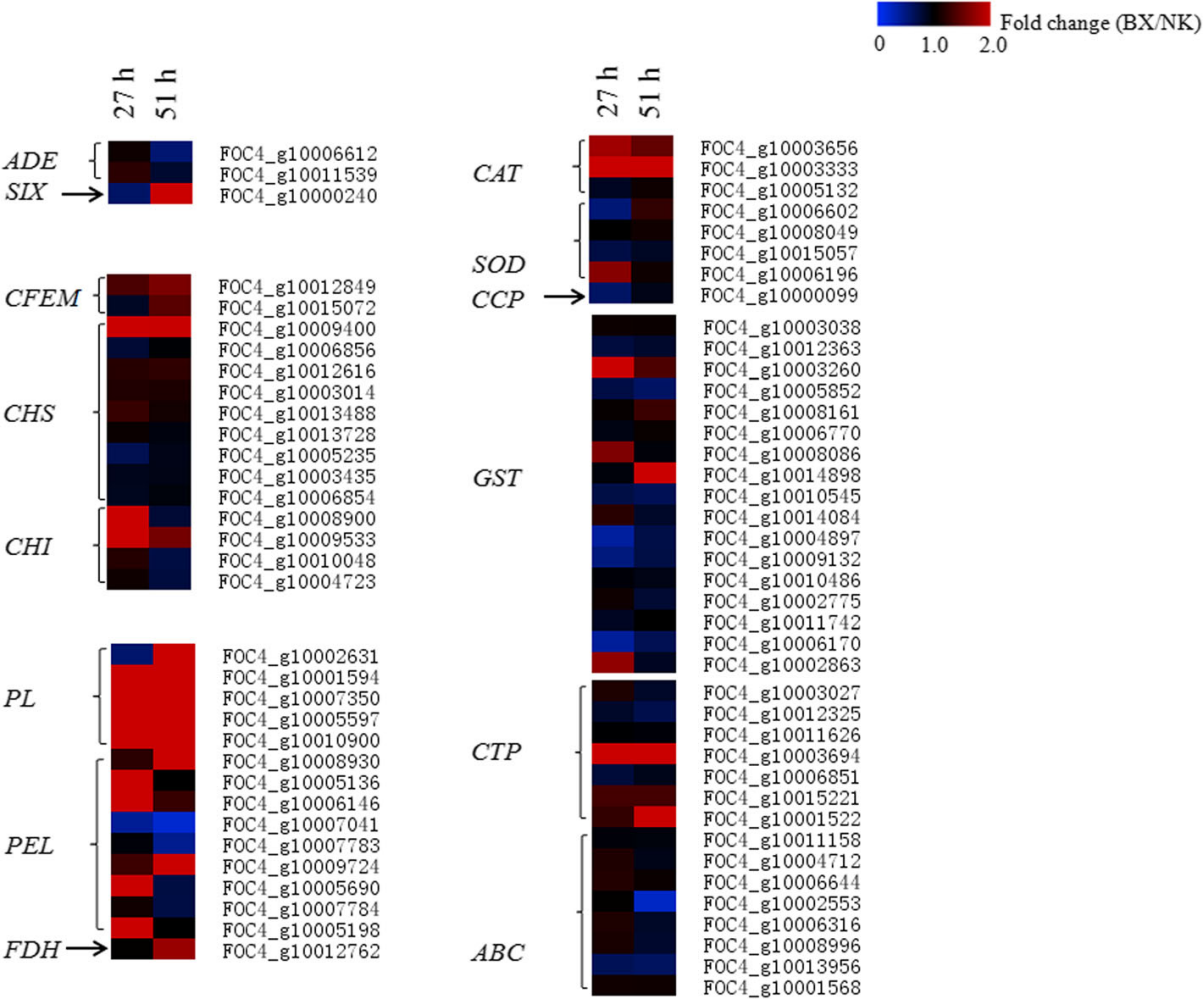
Expression fold change of fungal genes related to the virulence of pathogen in both banana cultivars. Columns from left to right represent the expression fold changes of genes from BX/NK at 27 hpi and BX/NK at 51 hpi. Each row represents one candidate gene. The expression fold change was represented by a color scale ranging from saturated blue for fold change = 0 to saturated red for fold change = 2.0 *ADE*: Alcohol dehydrogenase transcriptional effector, *SIX*: Secreted in xylem protein; *CFEM*: CFEM domain-containing proteins, *CHS*: Chitin synthase, *CHI*: Chitinase, *PL*: Pectin lyase, *PEL*: Pectate lyase, *FDH*: Formate dehydrogenase, *CAT*: Catalase, *SOD*: Superoxide dismutase, *CCP*: Cytochrome c peroxidase, *GST*: Glutathione S-transferase, *CTP*: Cytochrome P450, *ABC*: ATP binding cassette superfamily

We defined putative functions for 6690 of the 10,777 fungal transcripts present in banana roots through BLASTx analysis. Among them, many fungal genes related to virulence, manipulation and adaptation to the host environment were highly induced in the infected banana roots. The fold change of gene expression levels in BX over those in NK was investigated furthermore (Fig. 6). For instance, both glucose-repressible alcohol dehydrogenase transcriptional effector (*ADE*) genes were highly induced in two hosts. The Secreted In Xylem (*SIX*) gene was more greatly induced in BX compared to that in NK at 51 hpi. Fungal gene encoding GPI-anchored common in fungal extracellular membrane (CFEM) domain-containing protein plays an important role during colonizing and infecting on hosts [24]. Two *CFEMs* were also greatly induced in the infected BX roots with the fold change (> 1) especially at 51 hpi. Most of fungal genes encoding chitin synthase (CHS) and chitinase (CHI) were induced in both banana hosts. Especially, FOC4_g10009400, 10,008,900 and 10,009,533 with the high fold change of above 2 were greatly stimulated in BX. Most of fungal genes encoding pectin lyase and pectate lyase (PL and PEL) that break down the pectin present in the plant cell wall, were significantly induced in BX with the high fold changes (> 2), except for FOC4_g10007041 and 10,007,783. The expression level of *FDH* encoding formate dehydrogenase that involved in the metabolism of the products of pectin degradation was also greatly induced in the infected BX at 51 hpi (Fig. 6).

During its colonization in banana, fungus expressed a number of genes involved in detoxification and stress tolerance [25]. For instance, catalase peroxidase (*CAT*), superoxide dismutase (*SOD*) and cytochrome c peroxidase (*CCP*) genes were distinctively expressed in both hosts. Furthermore, two *CAT*s were significantly stimulated from BX, indicating that the fungus mounted more defenses in BX than in NK during the early infection stage. In addition, seventeen glutathione S-transferase (GST) family members, which combine glutathione with an electrophilic substance, and seven cytochrome P450 (CTP) members, which are indispensable for pathogens adapting to a hostile environment [26], were highly expressed in both hosts (Fig. 6). The fungus also induced many transporter genes to facilitate the infection of the plant. For instance, the ATP binding cassette superfamily (ABC) transporters, which mainly function in pathogen defense and transport of virulence factors [27], were also induced in both cultivars. The transcriptome data of all fungal genes were shown in Additional file 11: Table S7.

### Evidence of the nutritional-acquisition strategy of Foc TR4 fungus

During the infection, fungus also utilizes compounds from xylem vessels through secreting some special proteinases. The presence of genes encoding subtilisin-like proteinase, serine and aspartic proteinases would help the fungus degrade plant proteins and utilize nutrients to survive in hosts (Table 2). For instance, genes encoding subtilisin-like proteinase and aspartic proteinase were strikingly expressed in both banana hosts. Furthermore, serine protease and L-asparaginase genes, which assimilates the degraded proteins and catalyzes the release of ammonia from serine and asparagine, were also found in both cultivars. Fungal sugar transporter and nitrogen assimilator genes had the similar expression levels in both infected banana roots, except that FOC4_g10013479 and FOC4_g10009495 had higher expression levels at 27 hpi in BX than those in NK. These proteinases and transporters might benefit fungus to obtain its nitrogen and carbon sources from plant tissue.

**Table 2:** Expression levels of fungal genes related to nutrition utilization from banana roots

Gene function	Transcript_ID	BX27h	BX51h	NK27h	NK51h
Subtilisin-like proteinase	FOC4_g10014107	1940.2	1355.6	1399.5	1362.8
Serine protease	FOC4_g10012388	22.7	10.5	28.3	30.7
	FOC4_g10001080	21.9	11.2	24.5	37.1
	FOC4_g10001782	24.9	12.5	22.3	25.6
	FOC4_g10006912	63.2	76.8	49.6	129.3
Aspartic proteinase	FOC4_g10009784	485.3	444.7	154.3	85.2
L-asparaginase	FOC4_g10015082	56.7	66.9	98.3	79.5
	FOC4_g10011536	34.4	35.5	49.6	26.8
	FOC4_g10006918	21.9	35.6	41.3	35.3
Sugar transporter	FOC4_g10013479	35.0	8.7	8.2	15.1
	FOC4_g10007234	29.25	14.33	23.01	14.88
Nitrogen assimilation transcription factor	FOC4_g10014275	55.8	55.4	65.4	64.2
	FOC4_g10001030	29.8	24.3	30.8	25.6
	FOC4_g10003160	19.2	18.1	16.2	21.9
	FOC4_g10007075	18.9	25.5	21.1	25.6
	FOC4_g10009495	48.1	29.6	26.6	42.2
	FOC4_g10005284	17.9	19.7	14.3	20.1

The expression level is defined as FPKM

The Foc genome was from DDBJ/EMBL/GenBank under the accession number of AMGQ00000000

### Potential function of fungal genes *CCP 1*

One of the earliest defense reactions activated in plant tissues in response to pathogen attack is the accumulation of reactive oxygen species (ROS) [23, 28]. Necrotrophic pathogens even stimulate H_2_O_2_ production [29, 30]. Catalase (*CAT*) from *septoria tritici* in wheat effectively eliminates H_2_O_2_ to maintain the intercellular redox balance [31]. We also verified that *CAT* was greatly related to the pathogenicity of Foc TR4 during its infection into banana roots [32]. Cytochrome c peroxidase (CCP) is an antioxidant enzyme in the mitochondria; it is a possible sensor in the oxidative stress response in fungi [33]; and it is also one of the key enzymes for camalexin biosynthesis in plants [34]. To seek the role of *CCP* named as *CCP1* in Foc TR4, wild type (WT), mutant (*ccp1*) and complemented fungal strains (Comp) were generated to study their colonization, development, and pathogenicity in banana plants.

The *ccp1* and the Comp strains were constructed through PCR overlap and homologous recombination. The *ccp1* strains carried a cassette of green fluorescent protein and hygromycin, and the Comp strains carried a neomycin cassette for their selection and verification (Additional file 12: Figure S5). The *ccp1* strains showed strong green fluorescence and the multiple PCRs indicated that *CCP1* was successfully knocked out from the strains (Additional file 13: Figure S6A, B, C and D). The Comp strains were verified by green fluorescence deletion and the PCR products of about 820 bp. The PCR products were amplified with the *CCP1*-specific primer pair F/R and the PCR product has the same sequence with that of *CCP1* (Additional file 13: Fig. S6).

**Fig. 7:**
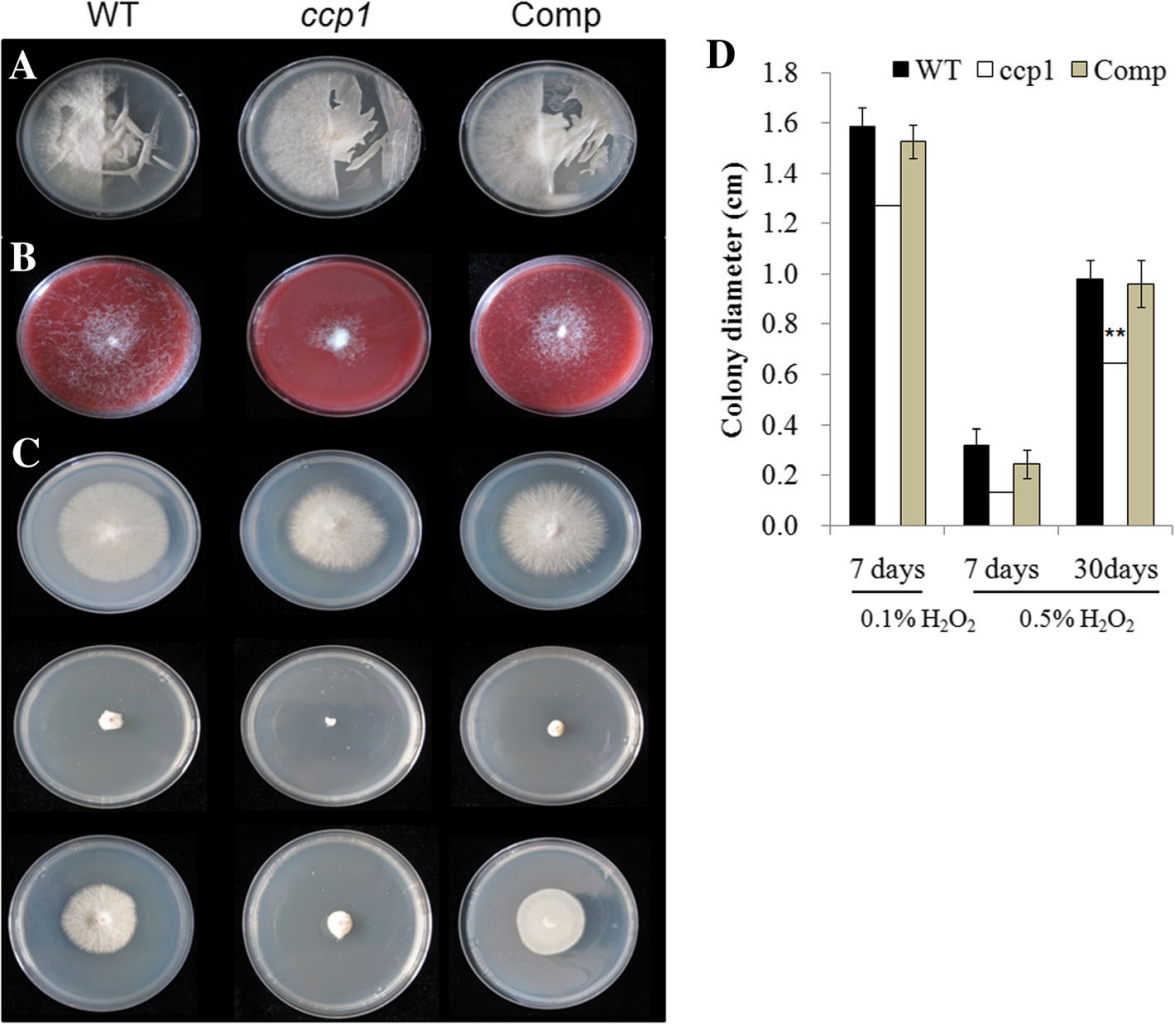
Penetration, cellulose utilization, and oxidative stress of different fungal strains. The WT, *ccp1*, and Comp fungal strains were generated from Foc TR4 VCG01213/16. **a** Fungal strains on PDA medium half-covered by cellophane after 7 d of growth at 28 °C. **b** Fungal strains on cellulose-Congo red medium after 7 d of growth at 28 °C. **c** Fungal strains on PDA medium with 0.1% H_2_O_2_ for 7 days (upper), with 0.5% H_2_O_2_ for 7 days (middle), and with 0.5% H_2_O_2_ for 30 days (lower) at 28 °C. **d** Colony diameter of all fungal strains under 0.1 and 0.5% H_2_O_2_ treatment. The error bars represent SE. Asterisks indicate significantly difference (** *P* < 0.01)

The morphology of fungi was no difference among WT, *ccp1*, and Comp strains grown on potato dextrose agar (PDA) medium at 28 °C for 4 d (Additional file 14: Figure S7). The cellophane is used to mimic the cell wall as a barrier to nutrient access to evaluate the penetration of fungus, as cutinase, cellulase, chitinase, and glucanase that benefit pathogenicity of fungus can penetrate the cellophane [35, 36]. The result showed that all strains could penetrate the cellophane, suggesting that *CCP1* should not impact the penetration of fungus into the plant cell wall. (Fig. 7a). The growth of the *ccp1* strains was greatly reduced after 7 days on cellulose-Congo Red medium, indicating that *CCP1* plays a role in cellulose utilization of fungus (Fig. 7b). WT, *ccp1*, and Comp strains showed greatly reduced growth at PDA with 0.1% H_2_O_2_ at 7 days (Fig. 7c). However, the *ccp1* strains were more significantly challenged with oxidative stress at 0.5% H_2_O_2_ compared to WT and Comp strains at 30 days (Fig. 7c and d), which was consistent with that *CCP* can change their oxidized state to eliminate O_2_^−^ or H_2_O_2_ [37, 38]. All strains hardly grew at 1% H_2_O_2_ even after 30 days (data not shown). In addition, all strains showed no differences when grown on PDA media containing 2 M NaCl or 2 M sorbitol, suggesting that *CCP1* might not be involved in osmotic stress or cell wall selective pressure for Foc TR4.

**Fig. 8:**
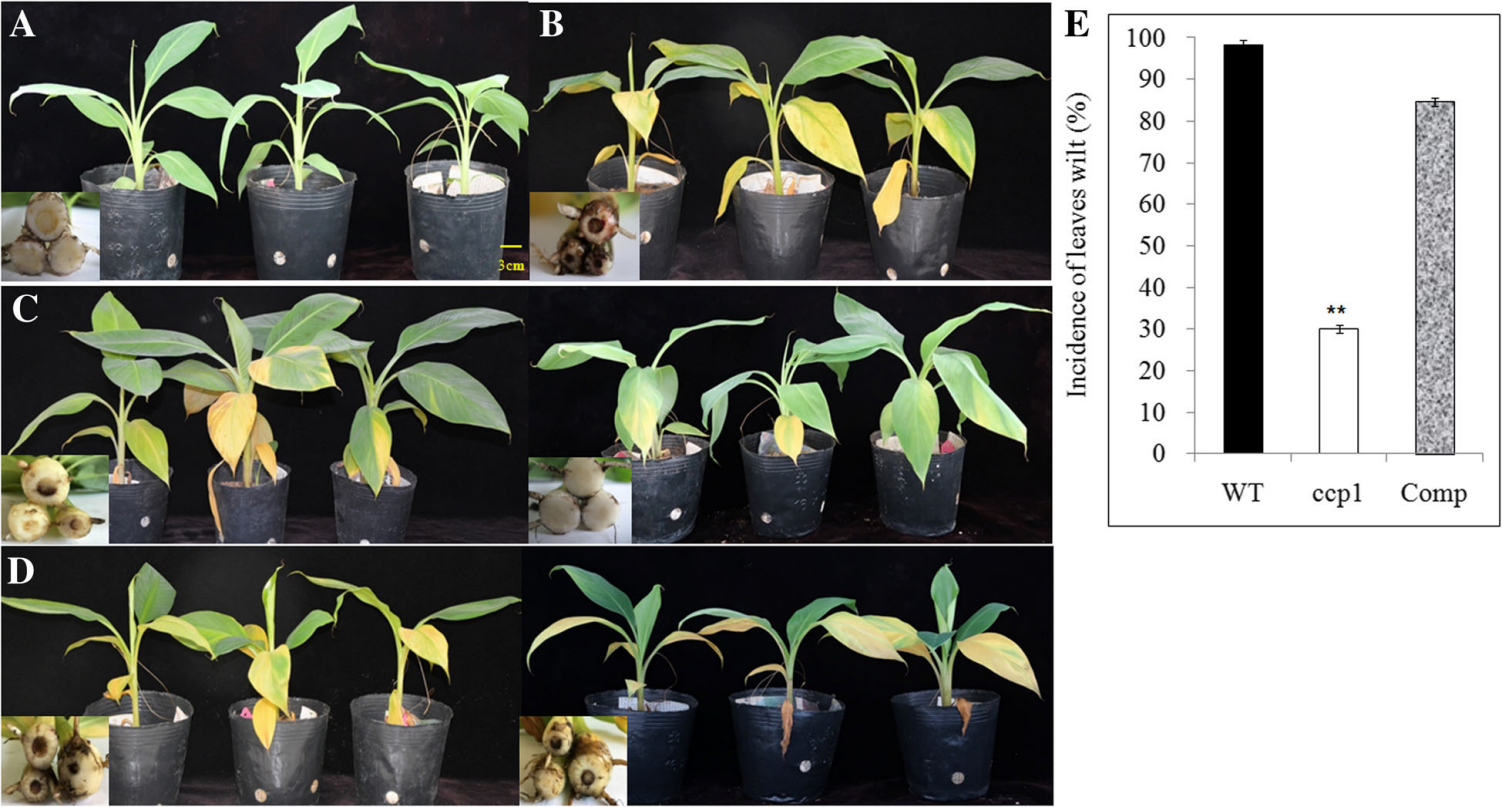
Phenotype and incidence of leave wilt of BX plantlets infected by different fungal strains for 45 days. Photographs were taken 45 days after inoculation. **a** Mock-inoculated banana plantlets. **b** Banana plantlets infected by the WT strains. **c** Banana plantlets infected by the *ccp1* strains. **d** Banana plantlets infected by the Comp strains. Each panel in the lower left is the transverse section of roots corresponding to the banana plantlets (from left to right). A total of 50 plants were inoculated for each Foc strain. The disease is diagnosed by wilt leaves and the brown spots in the roots. **e** The incidence of leaves wilt in banana plants infected by the WT, *ccp1* and Comp fungi strains. The error bars represent SE. Asterisks indicate significantly difference (** P < 0.01) through Student’s test

Furthermore, WT, *ccp1* and Comp strains were used to inoculate BX banana plantlets. The results displayed that the mock-inoculated banana plantlets showed green leaves and healthy roots (Fig. 8a). By contrast, all banana plantlets infected by either WT or Comp strains showed the typical yellowed-leaf phenotype and brown spots in roots at 45 days (Fig. 8b and d). The banana plantlets infected by the *ccp1* strains had only 30% disease incidence (Fig. 8c) that was significantly lower than those inoculated by WT and Comp strains (*p* value < 0.01) (Fig. 8e), suggesting that *CCP1* might play an important role in fungal pathogenicity.

## Discussion

Plants and pathogens constantly compete for survival, and the physiological and molecular change results in dynamic but regulated alterations in gene expression occurring in both organisms. In this respect, we present a dual high throughput RNA-seq on banana roots and Foc TR4 to unveil the dynamic changes in both species. It will provide a broader understanding of the banana defense response triggered by Foc TR4, or identify a potential pathogenic mechanism of Foc TR4 in both hosts with similar genetic background.

Since the transcriptomic profile reflects the culture condition and the treatment method, the test tissue and the fungi are crucial to this dual RNA-seq approach. In our research, the wounded banana roots wrapped with fungal agar block had more hyphea or conidia than those dipped into fungal suspension [13]. Our infection method resulted in some different expression profiles of genes from the previous researches [11, 12, 14]. For example, the number of up-regulated DEGs was more than that of the down-regulated DEGs in BX at both time points, while it showed the contrary in BX with roots soaking at 3 days after infection [11]. In addition, more up-regulated DEGs were found in NK at 27 hpi than in BX, although they were contrary at 51 hpi. Based on our dual RNA transcriptomic analysis, we try to present a model that shows the major molecular metabolism occurring in the test host and fungi (Additional file 15: Fig. S8 and data mainly from Additional files 3 and 16: Table S2 and S8). Briefly, the DEGs associated with glycolysis and glyoxylate cycle were widely involved in the defense response in the infected banana. Furthermore, the higher fold changes of these genes in the two pathways were found in NK than in BX at 27 hpi. Most DEGs required for the tricarboxylic acid cycle (TCA) were up-regulated only in the infected NK at 27 hpi, indicating that energy consumption was accelerated in the less susceptible cultivar. Together, many genes encoding trehalose phosphate phosphatase were more highly up-regulated in NK than in BX at 27 hpi, and a shift in metabolism could provide extra energy for banana plants to maintain growth and metabolism. These increasing in metabolism in NK at 27 hpi indicated that more energy would be available to resist the biotic stress at the infection early stage. However, it was likely that the glycerol metabolism were inhibited in NK due to more down-regulated genes at 27 hpi than in BX. Curiously, most of DEGs committed into the fatty acid degradation were only found in BX at 27 hpi. Based on our transcriptome, DEGs with the degradation of carbohydrates, including sucrose synthase, trehalose phosphate phosphatase, and α-amylase, were induced in both banana cultivars.

In addition, SA and JA are important signal molecules during the plant response to biotic and abiotic stresses [39, 40]. In our study, SA and JA were more greatly induced in the infected NK with the higher gene fold change than in the infected BX at 27 hpi. It suggested that SA and JA might be more early induced in the infected NK than in the infected BX. On the contrary, the fold change of DEGs involved in AUX and ABA metabolism showed higher in BX than in NK at 27 hpi. Phytohormone had cross-talk during modulating disease respond in plant [41]. The emerging evidence verifies that auxin is widely involved in plant disease susceptibility [42]. *GH3* increases the combination of IAA with amino acids to inactivate IAA, which stimulates the expression of *PR-1* and *CBF* in response to stress [43]. The levels of ABA are usually negatively associated with plant resistance due to its antagonistic interaction with the SA signaling pathway [44–46]. The induced DEGs related to ABA metabolism might contribute to the lower concentration of SA in BX than in NK.

The defense-related genes encoding PR1, thaumatin-like proteins (PR-5) peroxidase (PR-9), and chitinase play important roles in pathogen-infected plants or challenge with virulence factors from pathogens [9, 47]. These genes were more highly induced in NK than in BX at 27 hpi, which might be intimately related to the resistance of NK at the early infection stage (Additional file 3).

It was obvious that the chitin and pectin metabolism in Foc TR4 from the infected BX were higher compared to those from the infected NK. Chitin is an essential structural component of fungal cell walls, and fungus has multiple classes of *CHS* (chitin synthase) that deacetylate chitin to chitosan. The roles of some fungal *CHS* genes have been investigated in *Candida albicans*, *Aspergillus fumigatus*, *Aspergillus nidulans* and *Wangiella dermatiditis* [48, 49]. In our manuscript, more *CHS* genes were found compared to the previous research [15], and they were distributed in six classes according to the phylogenetic analysis [50, 51] (Additional file 17: Figure S9). It was notable that FOC4_g10006856 was clustered with FoCHSV that was required for pathogenesis of *Fusarium oxysporum* [52, 53], and it had the highest expression level (mean FPKM > 600 in BX and > 450 in NK) among all *CHS* genes. PL (pectin lyase) and PEL (pectate lyase) are the first two enzymes secreted by fungal pathogens when they directly degrade plant cell walls [54–56]. The high expression levels of *PL* and *PEL* genes might mean higher pathogenicity in the infected BX than in the infected NK.

Meanwhile, many fungal genes benefitting Foc TR4 infection and colonization were greatly induced in the infected banana roots. For instance, fungal effectors, defined as pathogen proteins and small molecules, facilitate infection and trigger defense response in fungi [57]. The Secreted In Xylem (*SIX*) gene has been identified as one family of effectors in *Fusarium oxysporum f sp. cubense* [58, 59]. The increasing expression level of *SIX* from 27 to 51 hpi (FPKM = 31 to 262.3) in the infected BX suggested that fungus might be more virulent to the susceptible BX, since it was hardly detected in the infected NK at 51 hpi. In agreement with this finding, 60 and 55% of the identified fungal genes were > 1-fold higher in BX than those in NK at 27 hpi and 51 hpi, respectively (data not shown). The higher expression levels of fungal genes in the BX might more suppress or postpone defense response in the susceptible BX than in the less susceptible NK. Many potential effectors including endoglucanase, elongation factor, and endopolygalacturonase found in our research needed to be investigated in the future (Additional file 18: Table S9). The fungal catalase, superoxide dismutase, cytochrome c peroxidase, glutathione S-transferase, and cytochrome P450 were widely induced in both hosts, which greatly contribute to remove active oxygen species from host, reduce the harm of reactive oxygen, and contribute to adaptation of fungi in host [60, 61].

The cytochrome c peroxidase (CCP) is a water-soluble heme-containing enzyme of the peroxidase family. It catalyzes the oxidation of organic substrates by H_2_O_2_ to resist peroxide-induce cellular stress in eukaryotes and prokaryotes [62–64]. In addition to its peroxidase activity, it acts as a sensor and signaling molecule to exogenous H_2_O_2_, which activates mitochondrial catalase activity [65]. *Campylobacter jejuni* carrying a mutation in the *Cj0020c* (*CCP*) gene are unable to effectively colonise chicks [66]. In our report, the mutant (*ccp1*) showed the greatly reduced virulence in banana and this is a unique result in our study, as *Cryotococcus neoformans* or the plant pathogen *Septoria tritici* lacking the *CCP* gene did not show diminished virulence [31, 67].

However, the fungal transcriptome also indicated that the lower mapped reads at 51 hpi than those at 27 hpi (Additional file 9: Table S5), which was inconsistent with the amount of hyphae or spores in the roots. It was reported that approximately 86.06 and 81.08% of genes in Foc TR4 were transcribed at vegetative stage and 48 h post inoculation [15], suggesting that the transcription of genes in Foc TR4 should be inhibited when facing to host. We speculated that the lower mapped reads at 51 hpi than at 27 hpi from Foc might be due to the inhibition from banana increasing immune response, which was consistent with the increased mapped reads from banana. However, the phenomenon might be temporary at the early infection stage, and it need to be investigated further.

Taken together, our research reported the intriguing interaction between *Fusarium oxysporum* f. sp. *cubense* and banana plant, which make wilt disease for further in-depth studies in future.

## Conclusion

1. Through the dual RNA-seq method, the dynamic transcript profiles at the early stages occurred in banana roots responding to Foc TR4 were explored in unprecedented perspectives. About 60 and 8% of the reads were mapped to the banana and fungal genome; however, 97% (35,276) of banana genes and 60% (6690) of the fungal genes were obtained in our research.

2. Infection with fungus triggers massive metabolic reprogramming in the banana roots using KEGG and GO analysis. More DEGs related to glycolysis, tricarboxylic acid cycle, and degradation of carbohydrates in NK at 27 hpi than in BX might be related to the less susceptibility of NK to Foc TR4.

3. The DEGs involved in the biosynthesis and metabolism of salicylic acid, jasmonic acid, auxin, and abscisic acid showed different expression changes between both cultivars, which contribute to the immune response of banana to Foc TR4.

4. The genes encoding the potential antivirulence, virulence, and effectors proteins were identified from Foc fungus. The high expression levels of these fungal genes in BX discovered that the fungus was more virulent to BX than NK, which might result in the susceptibility of BX to Foc TR4.

## Methods

### Plant growth conditions and fungus inoculation

The susceptible BX and the less susceptible NK of banana (*Musa acuminata*) cultivars were from the Tropical Crop Germplasm Research Institute, Chinese Academy of Tropical Agricultural Sciences (Danzhou city, Hainan, China). Tissue culture-derived plantlets were grown in pots under 16-h light/8-h dark at 28 °C with 100 μmol m^− 2^ s^− 1^ light intensity for 90 days. The Foc TR4 strains (VCG01213/16) isolated from Hainan in China by Dr. Junsheng Huang (Environment and Plant Protection Institute, Chinese Academy of Tropical Agricultural Sciences, Haikou, China) were used in our research. The fungus was transformed with GFP according to the reported method [21] and then used for the infection processing detection in banana roots. The GFP-expressing Foc TR4 was newly transferred to the potato dextrose agar (PDA) media with 100 μg/ml hygromycin B 4 to 5 days before plant inoculation.

The root epidermis was artificially damaged (about 0.5 cm^2^) and then covered with a freshly prepared Foc agar block of about 0.5 cm^2^ for fungus inoculation [24]. The treated banana roots above 0.5 cm of the infection point were observed with a laser confocal microscope (OLYMPUS, FV10-ASW) using GFP filters [13].

The number of hyphea or conidia in the vascular tissue was counted through the green fluorescent light points in an intact root transverse section. Six banana plants with 4 infected roots for every treatment samples and a total of about 60–80 roots sections were obtained from three replications. The statistical significance between different samples was carried out using Student’s test.

### Plant sampling and sample sequencing

About 10 roots of three banana plants inoculated by wild Foc TR4 for every treatment were collected at 27 h and 51 h post inoculation (hpi), respectively. RNA (3 μg) was extracted from the treated parts of banana roots using an RNA extraction kit (OMEGA, USA). Poly (A) -RNA enrichment and a strand-specific RNA-seq library were prepared using the NEBNext Ultra™ Directional RNA Library Prep Kit for Illumina (NEB, USA) following manufacturer’s recommendations. Library quality was assessed on the Agilent Bioanalyzer 2100 system (Agilent Technologies, USA). Libraries were sequenced on an Illumina Hiseq 2500 platform with 125-bp paired-end reads.

### Read mapping and gene expression quantification

After removing low-quality sequences containing uncalled bases (Ns), Bowtie v2.0.6 [68] and TopHat v2.0.9 [69] were used to align the RNA-seq reads against 36,542 gene models from *M. acuminata* (version 2, https://banana-genome-hub.southgreen.fr/) and 18,037 genes models from *Foc* (https://www.ncbi.nlm.nih.gov/nuccore/AMGQ00000000). Cuffdiff (v2.2.1) was used to calculate FPKMs (fragments per kilobase of transcript per million mapped reads) and to determine differential expression in each sample [70]. Hierarchical clustering for banana and fungal genes was performed with MultiExperiment Viewer (MeV) (version 4.8.1) from the TM4 suite (http://www.tm4.org) [71]. Genes were clustered using the Euclidean Distance measure and the complete linkage clustering method. PCA was conducted using the prcomp command with default parameters in the R software package. The transcriptome datasets are available at the NCBI Sequence Read Archive (SRA) with the accession number from SRR8661622 to 45. The expression levels of all banana plant transcripts are shown in Additional file 19: Table S10.

### Gene functional classification

The functions of genes were annotated using BLASTx with an e-value threshold of 10^− 5^ against the NCBI non-redundant (NR) protein database, the KEGG (Kyoto Encyclopedia of Genes and Genomes) protein database, COGs (NCBI phylogenetic classification of proteins encoded in complete genomes), and the Swiss-Prot database.

### GO and KEGG enrichment analyses on differentially expressed genes

Gene Ontology (GO) enrichment analysis of differentially expressed genes was conducted using the GOseq R package. GO terms with corrected *p*-values less than 0.05 were considered significantly enriched. KOBAS software was used to test the statistical enrichment of DEGs in the Kyoto Encyclopedia of Genes and Genomes (KEGG) pathways.

### Comparing RNA-seq and qPCR results

The qPCR assays were performed to confirm RNA-seq results by an independent technique (Additional file 2: Table S1). The expression levels of 30 banana and 10 Foc genes were analyzed by qPCR from the RNA sampled above. The qPCR experiments were conducted on a Step One Real-Time PCR system (Applied Biosystems) using SYBR Green I (Takara, Japan). Each reaction was performed in a final volume of 20 μl, containing 10 μl of 2 × SYBR Green PCR Master Mix (Takara, Japan), 200 nM each gene-specific primer, and 50 ng cDNA template. No-template reactions were included as negative controls for each set of primers used. The thermal cycling conditions were 95 °C for 30 s, followed by 40 cycles of 5 s at 95 °C, 20 s at 58–62 °C depending on primer melting temperature, and 20 s at 72 °C, with fluorescence detection at the end of each cycle. The amplification of a single product per reaction was confirmed by melting curve analysis. All reactions were performed in technical triplicates. Banana data were normalized using two reference genes that showed little variation in the RNA-seq analysis (*Actin* and glyceraldehydes-3-phosphate dehydrogenase 2, *GAPDH*). Expression levels of fungal genes were given in relation to the fungal *β-actin* and *IF3b* (transcription initiation factor) genes. Primers used in these experiments were designed by Primer Premier 6.0 software.

### Construction of mutant and complementation cassettes targeting cytochrome c peroxidase (*CCP1*)

The mutant of *CCP1* deletion (*ccp1*) was performed by homologous recombination. In brief, the 5′- and 3′-flanking sequences of the *CCP1* gene were amplified using the genomic DNA as a template with primer pairs F1/F2 and F3/F4, respectively (Additional file 12: Figure S5, upper). The hygromycin (HYG) and green fluorescent protein (GFP) cassette was amplified with primer pairs M1/M2 from the binary vector pCT74. Three respective products were ligated through three rounds of PCR for the *ccp1* strain construction.

For the mutant complementation (Comp) construction, the ORF of the *CCP1* gene was amplified with the 5′-flanking sequences using primer pair F1/CR from the Foc genomic DNA. The product was overlapped with a neomycin cassette (amplified using primers C1/C2), and the 3′-flanking sequences (amplified using primers CF3/F4) through PCR using primer pairs F1/F4 (Additional file 12: Figure S5, lower). Briefly, in the first round, three fragments were amplified and extracted. In the second round, all three amplified products with the same quantity of total DNA (> 800 ng) were amplified using LA Taq polymerase (Takara, Japan) without primers to produce the full-length fusion PCR products. In the third round, primers F1 and F4 were used to yield the mutant and the complementation construction. All primers are shown in Additional file 20: Table S11.

The first- and third-round amplification reactions consisted of 35 cycles of 10 s at 98 °C, 5 s at 55 °C, and 72 °C, 25 s. The second-round reactions consisted of 31 cycles of 5 min at 94 °C, 30 s at 94 °C, 55 °C, 30 s and a final extension of 7 min at 72 °C. The final reaction was the same with the first round except for the 30 s cycle length at 72 °C due to the longer products.

### PEG-mediated transformation of Foc protoplast

About 600 mg of fresh-cultured hyphae from Foc TR4 was catalyzed with 5 ml of mixed lysate (40 mg driselase, 40 mg lyticase and 20 mg snailase in 2 ml NaCl (0.8 M)) at 28 °C, for 2–3 h. After cleavage, the precipitate was collected at 2800 rpm for 10 min, washed with 40 ml of STC (1.2 mM sorbitol, 50 mM CaCl_2_, 10 mM Tris, pH 7.5), centrifuged at 3200 rpm for 10 min, and then redissolved in 200 μl of STC for transformation.

Each fusion PCR product (4 μg), i.e. the mutant or complementation cassette, was added into 200 μl STC containing catalyzed Foc TR4, and let to stand for 20 min. After that, the solution was added with 1.25 ml PTC (1000 mg PEG4000 in 2 ml STC) under gently shaking, and then again set for 20 min. The mutant and complemented strains grew on PDA with hygromycin (100 μg/ml) and neomycin (100 μg/ml), respectively, at 28 °C for 4–6 d.

### Analysis of *CCP1* gene function in the *ccp1* and comp strains of Foc

The penetration abilities of all strains were checked by growth on a cellophane membrane [72] that covered a semicircle of the pathogen plaque, while the other half remained uncovered, at 28 °C for 5–7 d.

Cellulose-Congo Red medium (Sangon, Shanghai, China) was used to track the utilization of cellulose by all strains [73]. Cellulose Congo Red Medium (25.3 g) and agar were dissolved into 1 L dH_2_O and autoclaved at 121 °C for 15 min.

Oxidative stress was applied by adding H_2_O_2_ to a final concentration of 0.1, 0.5%, or 1% to the PDA medium.

Osmotic stress was applied at two concentrations, 1 M or 2 M NaCl, in the PDA medium.

Cell wall selection pressure was tested with two concentrations of sorbitol (1 M and 2 M) in the PDA medium.

### The phenotype of banana infected by the *ccp1* strains

Healthy roots of the BX were inoculated by all strains according to our previous method [24]. The banana plants were planted as description before for 45 d. We collected 50 banana plants per treatment for phenotypic analysis. The incidence of wilt was the ratio of plants with wilt leaves and brown spots in pseudostem to all treated plants. Statistical significance between treatments was carried out using Student’s test.

## Additional files

Additional file 1: **Figure S1.** The morphology of BX and NK banana infected by Foc TR4 at 45 days. Chlorosis of the diseased leaves in BX (A, left) was consistent with the brown spot in pseudostem (B, left) at 45 days after Foc TR4 infection. Green leaves and healthy roots present in the less susceptible cultivar NK (A and B, right), (TIF 9758 kb)

Additional file 2: **Table S1**. Verification of RNA-seq results by qRT-PCR in banana roots infected by Foc TR4. (XLS 35 kb)

Additional file 3: **Table S2.** The expression levels of genes related to plant-pathogen interaction in banana roots. (XLS 41 kb)

Additional file 4: **Figure S2.** Metabolic pathways of the DEGs from banana as determined by GO analysis. The DEGs were obtained with the threshold of |Log_2_^(inoculated /mock-inoculated)^ | ≥1 and *q* value < 0.05 in banana. (A) The up-regulated DEGs, (B) the down-regulated DEGs. Purple, BX 27 hpi; Pink, NK at 27 hpi; Green, BX at 51 hpi; Red: NK at 51 hpi. (TIF 4327 kb)

Additional file 5: **Figure S3.** KEGG analysis on the DEMGs in banana roots from two mock-inoculated cultivars. The DEMGs were obtained with the threshold of |Log_2_^(BXMK/ NKMK)^ | ≥1 and *q* value < 0.05. MK: Mock-inoculated. Red, the up-regulated genes at 27 hpi; Blue, the down-regulated genes at 27 hpi; Pink, the up-regulated genes at 51 hpi; Black, the down-regulated genes at 51 hpi. (TIF 1401 kb)

Additional file 6: **Figure S4.** Metabolic pathways of the DEMGs from two mock-inoculated cultivars by GO analysis. The DEMGs were obtained with the threshold of |Log_2_^(BXMK/NKMK)^ | ≥1 and *q* value < 0.05. MK: Mock-inoculated. (A) The up-regulated DEMGs at 27 hpi; the down-regulated DEMGs at 27 hpi were hardly clustered under this threshold. (B) The DEMGs at 51 hpi. Purple, the up-regulated DEMGs; Black, the down-regulated DEMGs. (TIF 4311 kb)

Additional file 7: **Table S3.** The verification of gene expression levels involved in phytohormone transduction in Fig. 4. (XLS 39 kb)

Additional file 8: **Table S4.** The expression levels of genes related to other phytohormone biosynthesis and metabolism in banana during Foc TR4 infection. (XLS 20 kb)

Additional file 9: **Table S5.** Sequencing Metrics of Foc TR4 from 24 RNA-seq libraries. (XLS 21 kb)

Additional file 10: **Table S6**. The differentially expressed Foc genes (DEFGs) during infection from banana roots. (XLS 18 kb)

Additional file 11: **Table S7.** The average expression abundance of fungal genes in Fig. 6. (XLS 36 kb)

Additional file 12: **Figure S5**. Construction of the mutant (*ccp1*) and the complemented (Comp) Foc strains. The capital A and B denote the 5′- and 3′-flanking sequences of *CCP1* gene. Construction of the mutant cassette and its homologous recombination into the genome of Foc TR4 (upper). The fragments A and B were amplified with primers pairs F1/F2 and F3/F4, respectively, from Foc genomic DNA. The HYG + GFP cassette, including the *HYG* ORF, the TrpC promoter for *HYG*, the *GFP* ORF and the ToxA promoter for *GFP*, was amplified using M1/M2 from the vector pCT74. Construction of the complementation cassette and its homologous recombination into the genome of the *ccp1* mutant strains (lower). The fragments A + CCP1 and B were amplified with primer pairs F1/CR and CF3/F4, respectively, from wild Foc TR4 genomic DNA. The NEO cassette, including the neomycin (NEO) ORF and its promoter, was amplified using C1/C2 from the vector pKOV21. Other primers labeled in this figure were used for the verification of mutant and complemented Foc strains in Additional file 13: Figure S6. (TIF 1019 kb)

Additional file 13: **Figure S6.** Verification of the mutant (*ccp1*) and the complemented (Comp) Foc strains. (A) Green fluorescence picture of mycelium from the mutant Foc strains (*ccp1*). (B) PCR amplification of the HYG + GFP cassette from the *ccp1* strains using primer pairs H1/H2. Lane 1 to 6: the *ccp1* strains and the fragments of about 490 bp were amplified; lane 7: wild type Foc; lane 8: the positive control (pCT74 vector as template); lane 9: the negative control (no template). (C) PCR amplification of *CCP1* gene from the *ccp1* strains using primer pairs F/R. Lane 1 to 6: the *ccp1* strains. The *CCP1* gene was completely knocked out in the *ccp1* strains of lane 2, 4 and 6. Lane 7: the wild type Foc TR4. Lane 8: The negative control (no template). (D) Verification of the inserted 5′- and 3′- flanking sequences in the *ccp1* strains. Lanes 1 to 6: The 5′-flanking fragments (A) (about 2.0 kb) PCR product using primer pairs F1/MF2 and the *ccp1* strains as templates. Lane 7: wild type Foc DNA as template. Lane 8: the negative control (no template). Lanes 9 to 14: The 3′-flanking fragments (B) (about 1.9 kb) PCR product using primer pairs MF3/F4 and the *ccp1* strains as templates; Lane 15: the wild type Foc DNA as template; Lane 16: the negative control. (E) Verification of the Comp strains. The PCR products using the primer pairs F/R. Lane 1 to 5: the Comp strains 1 to 5 as templates. Lane 6: the negative control with the *ccp1* strain 6 as template. Lane 7: the positive control with the wild type as template. (TIF 14632 kb)

Additional file 14: **Figure S7.** The phenotype of wild type (WT), *ccp1*, and Comp fungal strains on PDA at 28 °C for 4–6 d. (TIF 7180 kb)

Additional file 15: **Figure S8.** A representative model of the interaction between different banana cultivars and Foc. (A) The response of BX to Foc TR4; (B) The response of NK to Foc TR4 CFEM: CFEM domain-containing proteins, CAT: Catalase, CCP: Cytochrome c peroxidase, GST: Glutathione S-transferase, ABC transporter: ATP binding cassette superfamily. (TIF 8095 kb)

Additional file 16: **Table S8.** The differentially expressed genes related to carbon metabolism in banana during Foc TR4 infection. (XLS 35 kb)

Additional file 17: **Figure S9.** Phylogenetic relationship of fungal chitin synthases. Sequences were taken from GenBank or genome projects. The neighbor-joining tree was constructed using clustal W program (http://clustalw.genome.jp/) and MEGA software version 5.0. The chitin synthase (CHS) are ScCHS1, 2 of *Saccharomyces cerevisiae*, NcCHS1, 2, and 3 of *Neurospora crassa*, CaCHS1, 2, and 3 of *Candida albicans*, BgCHS2 of *Blumeria graminis*, MgCsm1 of *Magnaporthe grisea*, FoCHSV of *Fusarium oxysporum*, CgCHSA of *Colletotrichum graminicola*, EdCHS5 of *Exophiala dermatitidis*, PbCHS4 of *Paracoccidioides brasiliensis*, AnCsmA of *Aspergillus nidulans*, AoCHSY and AoCHSZ of *Aspergillus oryzae*, and AnCsmB of *Aspergillus nidulans*. The sequence of AnCHSE,AfCHSC and AfCHSG were obtained from reference [58, 59]. (TIF 4315 kb)

Additional file 18: **Table S9.** The putative virulence associated genes in the banana fungal pathogens Foc TR4. (XLS 43 kb)

Additional file 19: **Table S10.** The expression levels of all banana transcripts in our research. (XLS 25512 kb)

Additional file 20: **Table S11.** The primers for the *ccp1* and Comp Foc strains construction. (XLS 28 kb)

## Data Availability

The sequences of all transcripts are available in the published banana genome data (version 2, http://banana-genome- hub.southgreen.fr/) and the raw data of transcriptome was uploaded in NCBI with the accession number from SRR8661622 to 45.
