# Supplementary figures and images for: Dual species transcript profiling during the interaction between banana (Musa acuminata) and the fungal pathogen Fusarium oxysporum f. sp. cubense
Source: BMC Genomics. 2019 Jun 24;20:519. doi: 10.1186/s12864-019-5902-z (PMC6591919; doi:10.1186/s12864-019-5902-z)

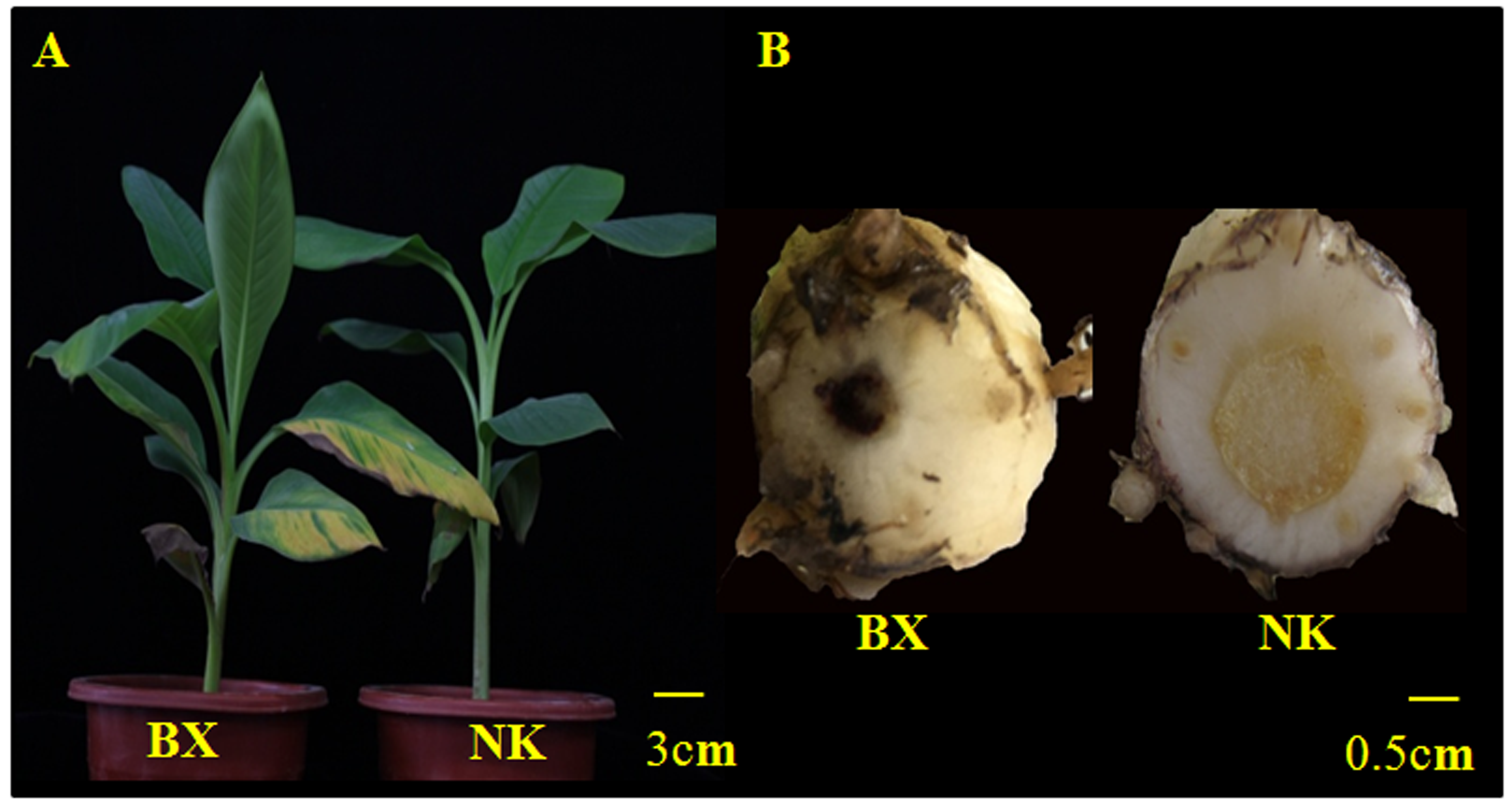

Supplement: Supplementary file 1 — Figure S1. The morphology of BX and NK banana infected by Foc TR4 at 45 days. Chlorosis of the diseased leaves in BX (A, left) was consistent with the brown spot in pseudostem (B, left) at 45 days after Foc TR4 infection. Green leaves and healthy roots present in the less susceptible cultivar NK (A and B, right), (TIF 9758 kb) [file 12864_2019_5902_MOESM1_ESM.tif]

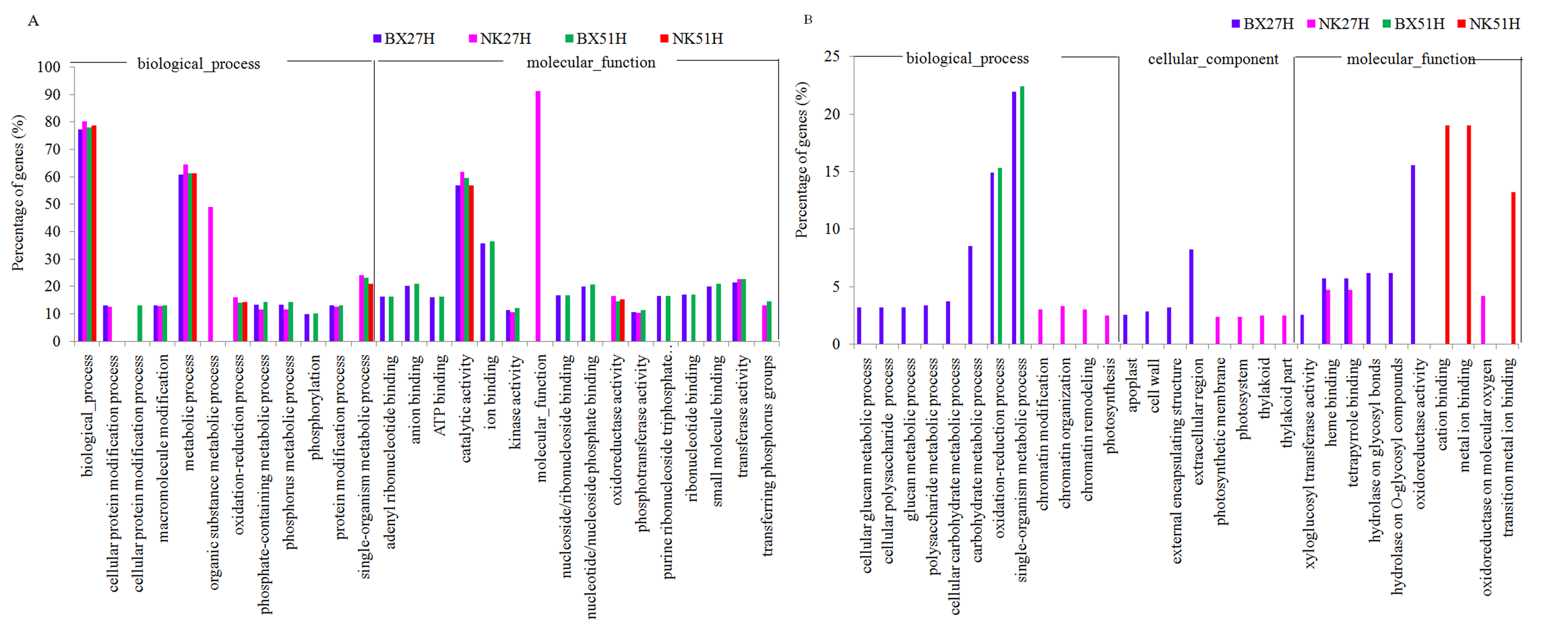

Supplement: Supplementary file 4 — Figure S2. Metabolic pathways of the DEGs from banana as determined by GO analysis. The DEGs were obtained with the threshold of |Log2 (inoculated /mock-inoculated) | ≥1 and q value < 0.05 in banana. (A) The up-regulated DEGs, (B) the down-regulated DEGs. Purple, BX 27 hpi; Pink, NK at 27 hpi; Green, BX at 51 hpi; Red: NK at 51 hpi. (TIF 4327 kb) [file 12864_2019_5902_MOESM4_ESM.tif]

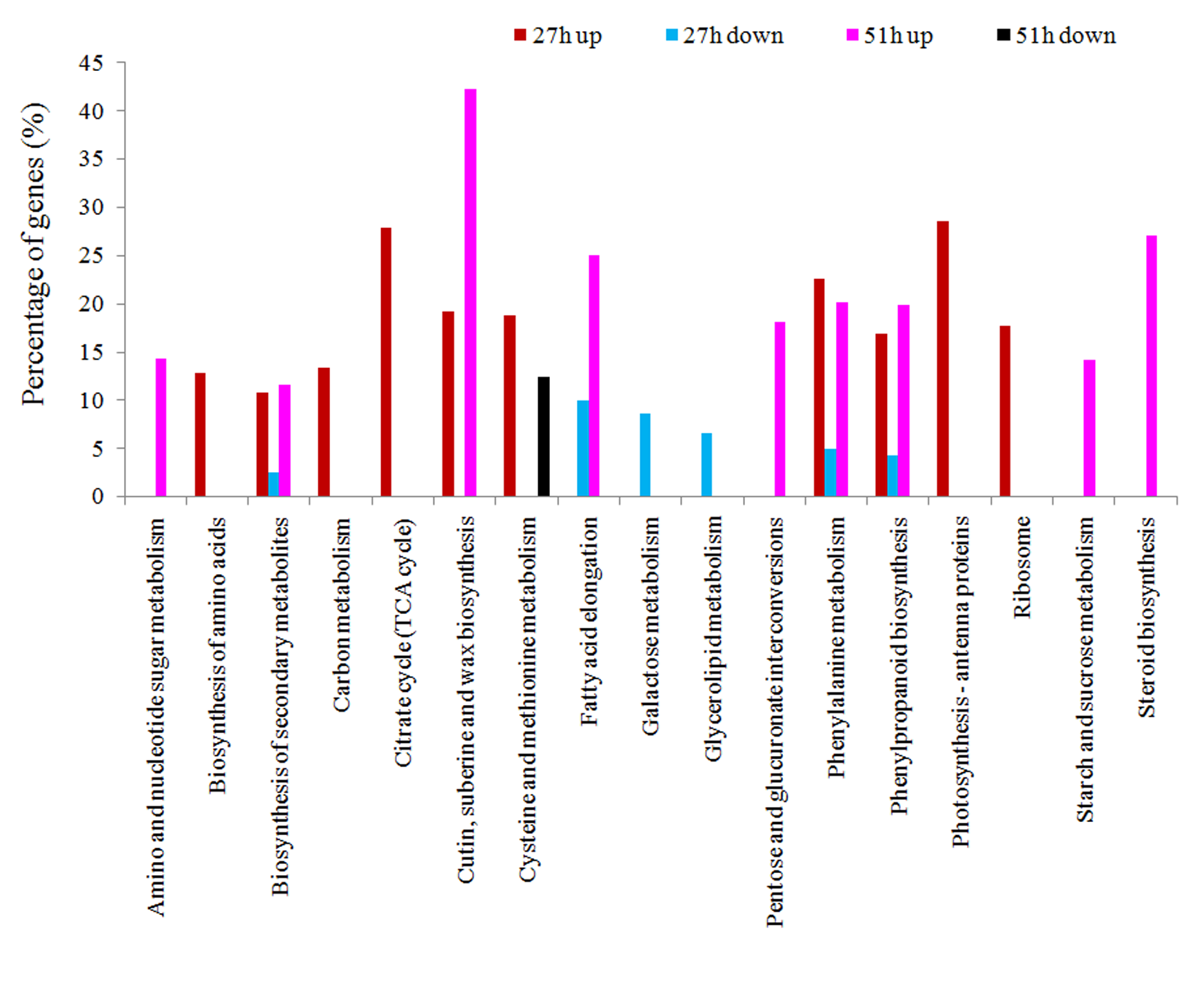

Supplement: Supplementary file 5 — Figure S3. KEGG analysis on the DEMGs in banana roots from two mock-inoculated cultivars. The DEMGs were obtained with the threshold of |Log2 (BXMK/ NKMK) | ≥1 and q value < 0.05. MK: Mock-inoculated. Red, the up-regulated genes at 27 hpi; Blue, the down-regulated genes at 27 hpi; Pink, the up-regulated genes at 51 hpi; Black, the down-regulated genes at 51 hpi. (TIF 1401 kb) [file 12864_2019_5902_MOESM5_ESM.tif]

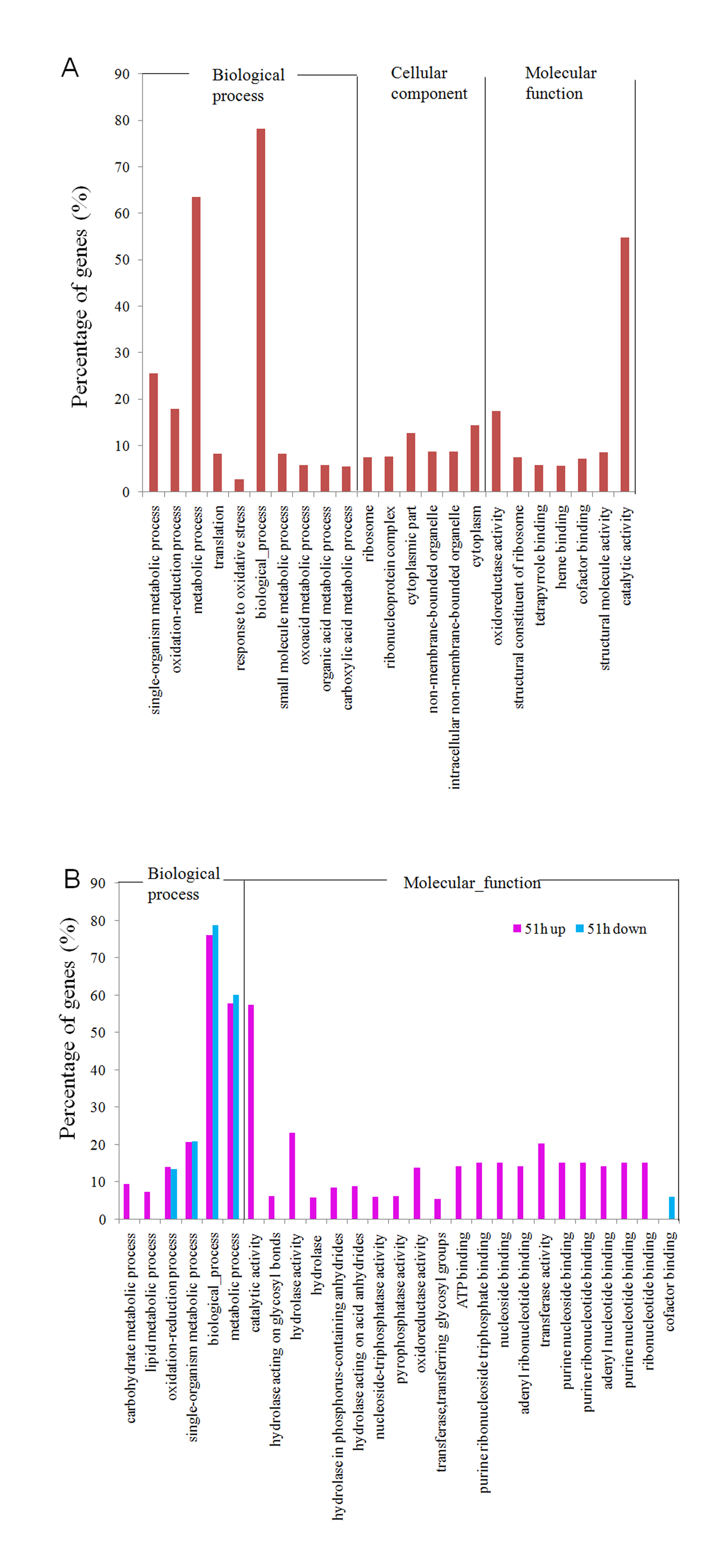

Supplement: Supplementary file 6 — Figure S4. Metabolic pathways of the DEMGs from two mock-inoculated cultivars by GO analysis. The DEMGs were obtained with the threshold of |Log2 (BXMK/NKMK) | ≥1 and q value < 0.05. MK: Mock-inoculated. (A) The up-regulated DEMGs at 27 hpi; the down-regulated DEMGs at 27 hpi were hardly clustered under this threshold. (B) The DEMGs at 51 hpi. Purple, the up-regulated DEMGs; Black, the down-regulated DEMGs. (TIF 4311 kb) [file 12864_2019_5902_MOESM6_ESM.tif]

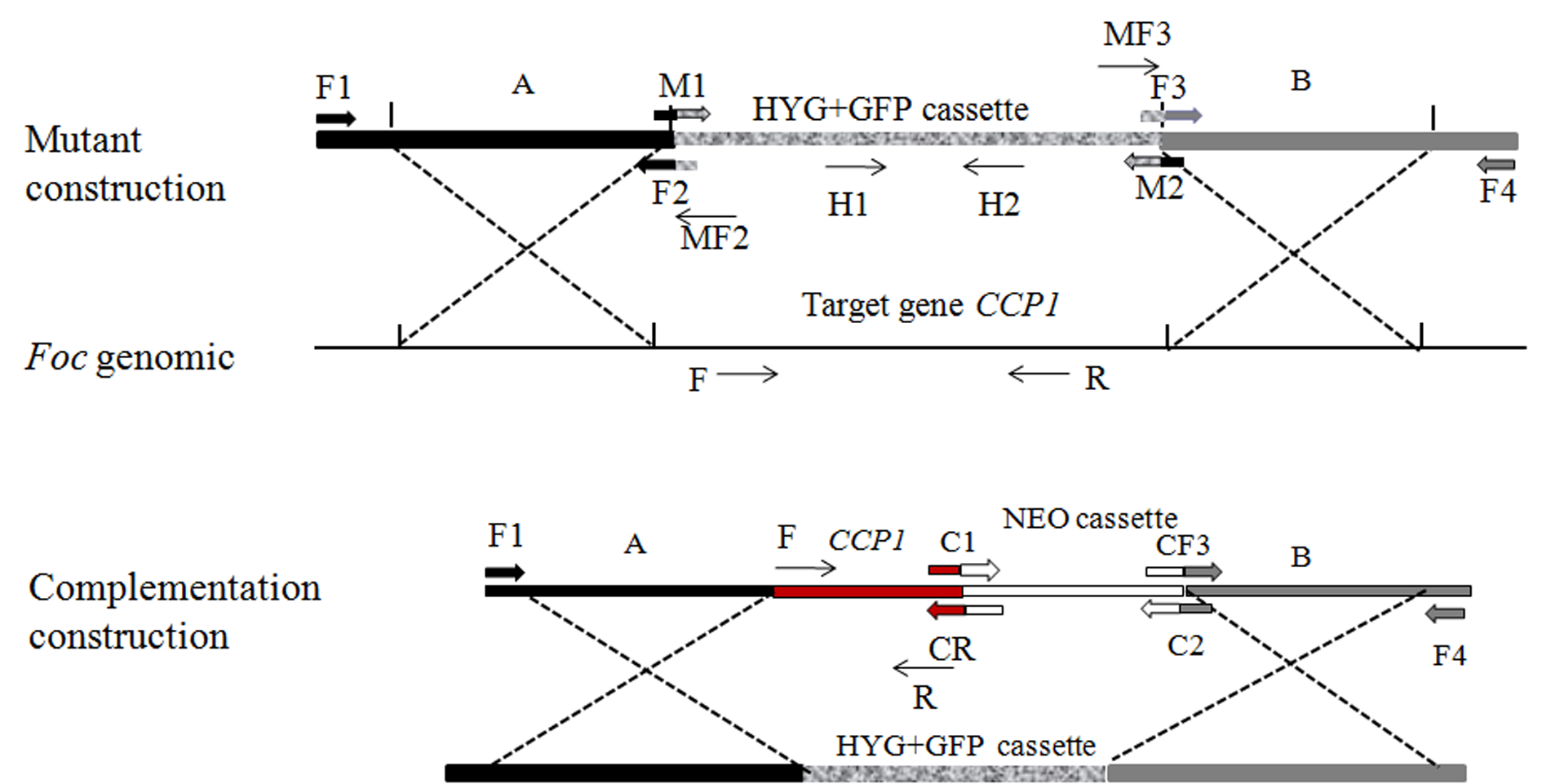

Supplement: Supplementary file 12 — Figure S5. Construction of the mutant (ccp1) and the complemented (Comp) Foc strains. The capital A and B denote the 5′- and 3′-flanking sequences of CCP1 gene. Construction of the mutant cassette and its homologous recombination into the genome of Foc TR4 (upper). The fragments A and B were amplified with primers pairs F1/F2 and F3/F4, respectively, from Foc genomic DNA. The HYG + GFP cassette, including the HYG ORF, the TrpC promoter for HYG, the GFP ORF and the ToxA promoter for GFP, was amplified using M1/M2 from the vector pCT74. Construction of the complementation cassette and its homologous recombination into the genome of the ccp1 mutant strains (lower). The fragments A + CCP1 and B were amplified with primer pairs F1/CR and CF3/F4, respectively, from wild Foc TR4 genomic DNA. The NEO cassette, including the neomycin (NEO) ORF and its promoter, was amplified using C1/C2 from the vector pKOV21. Other primers labeled in this figure were used for the verification of mutant and complemented Foc strains in Additional file 13: Figure S6. (TIF 1019 kb) [file 12864_2019_5902_MOESM12_ESM.tif]

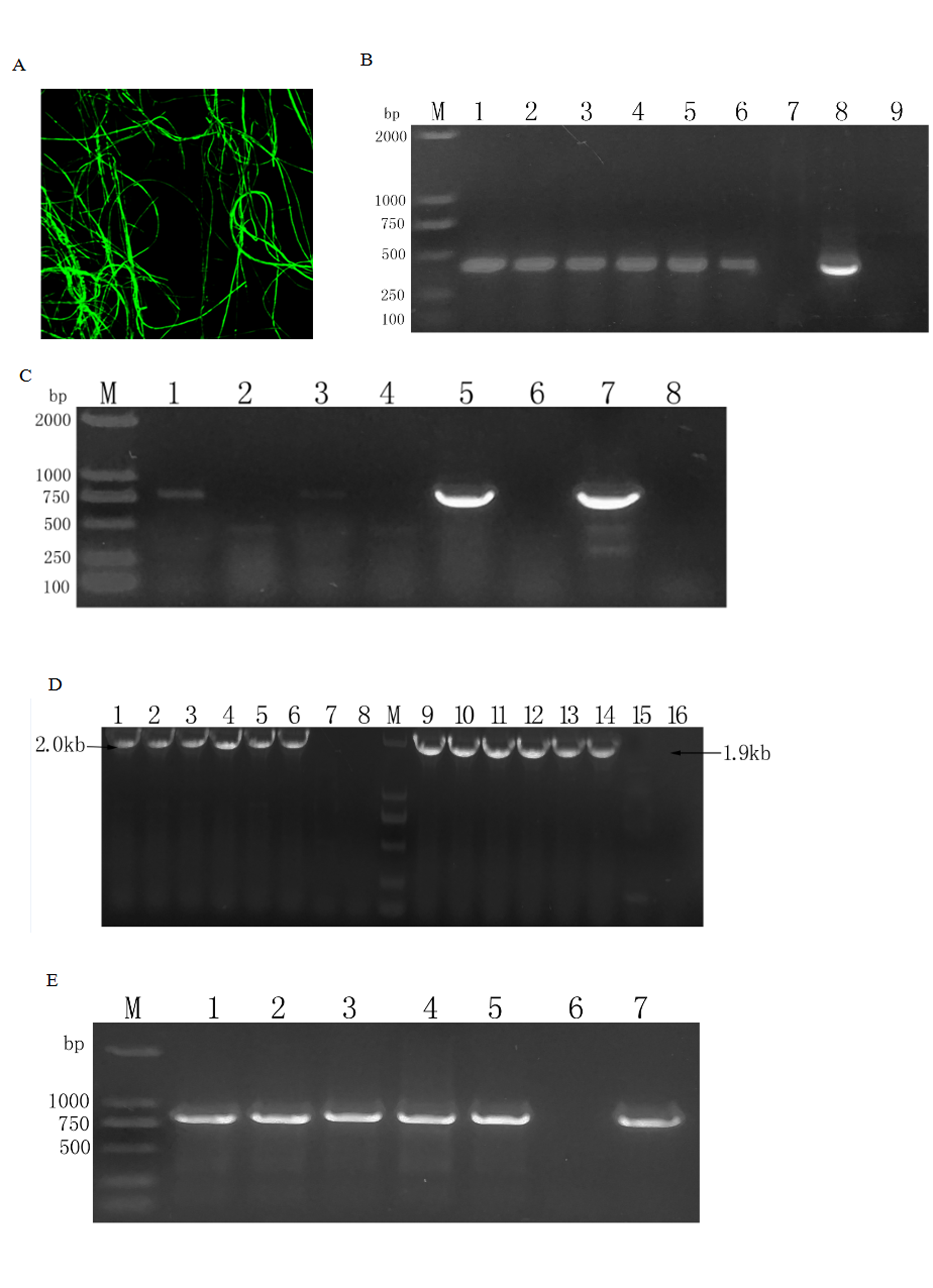

Supplement: Supplementary file 13 — Figure S6. Verification of the mutant (ccp1) and the complemented (Comp) Foc strains. (A) Green fluorescence picture of mycelium from the mutant Foc strains (ccp1). (B) PCR amplification of the HYG + GFP cassette from the ccp1 strains using primer pairs H1/H2. Lane 1 to 6: the ccp1 strains and the fragments of about 490 bp were amplified; lane 7: wild type Foc; lane 8: the positive control (pCT74 vector as template); lane 9: the negative control (no template). (C) PCR amplification of CCP1 gene from the ccp1 strains using primer pairs F/R. Lane 1 to 6: the ccp1 strains. The CCP1 gene was completely knocked out in the ccp1 strains of lane 2, 4 and 6. Lane 7: the wild type Foc TR4. Lane 8: The negative control (no template). (D) Verification of the inserted 5′- and 3′- flanking sequences in the ccp1 strains. Lanes 1 to 6: The 5′-flanking fragments (A) (about 2.0 kb) PCR product using primer pairs F1/MF2 and the ccp1 strains as templates. Lane 7: wild type Foc DNA as template. Lane 8: the negative control (no template). Lanes 9 to 14: The 3′-flanking fragments (B) (about 1.9 kb) PCR product using primer pairs MF3/F4 and the ccp1 strains as templates; Lane 15: the wild type Foc DNA as template; Lane 16: the negative control. (E) Verification of the Comp strains. The PCR products using the primer pairs F/R. Lane 1 to 5: the Comp strains 1 to 5 as templates. Lane 6: the negative control with the ccp1 strain 6 as template. Lane 7: the positive control with the wild type as template. (TIF 14632 kb) [file 12864_2019_5902_MOESM13_ESM.tif]

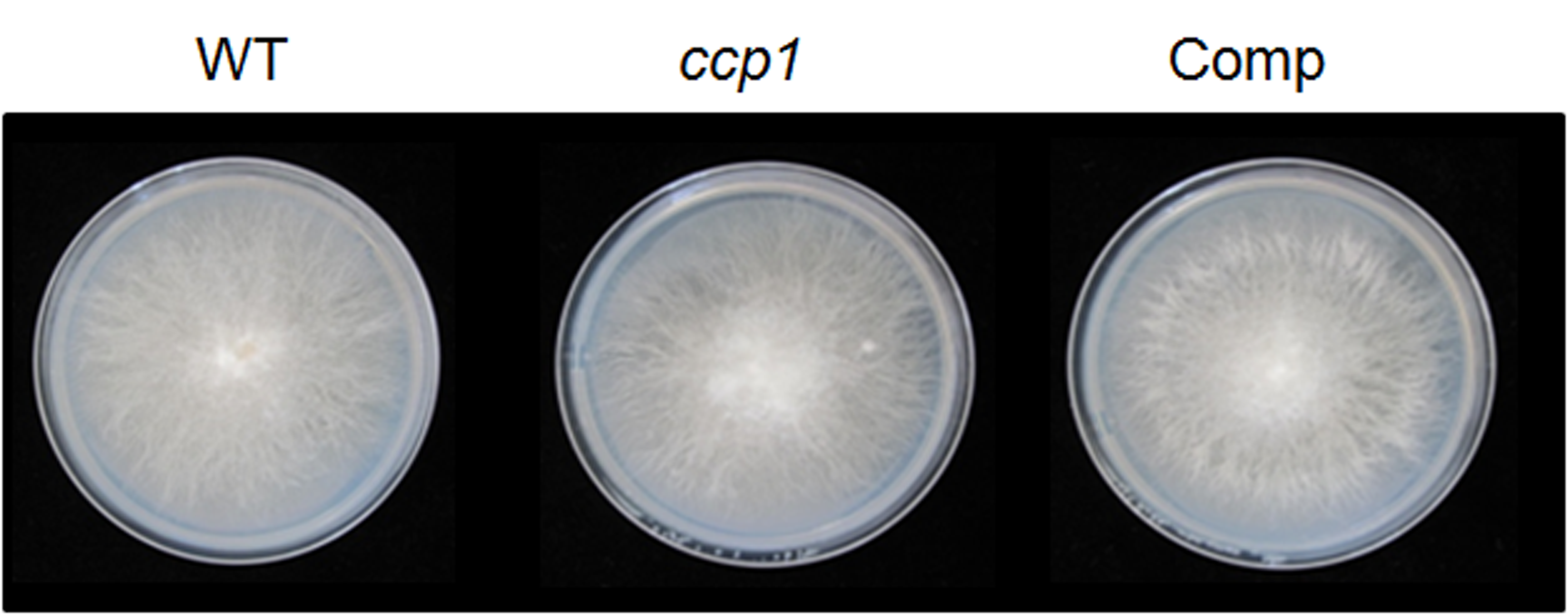

Supplement: Supplementary file 14 — Figure S7. The phenotype of wild type (WT), ccp1, and Comp fungal strains on PDA at 28 °C for 4–6 d. (TIF 7180 kb) [file 12864_2019_5902_MOESM14_ESM.tif]

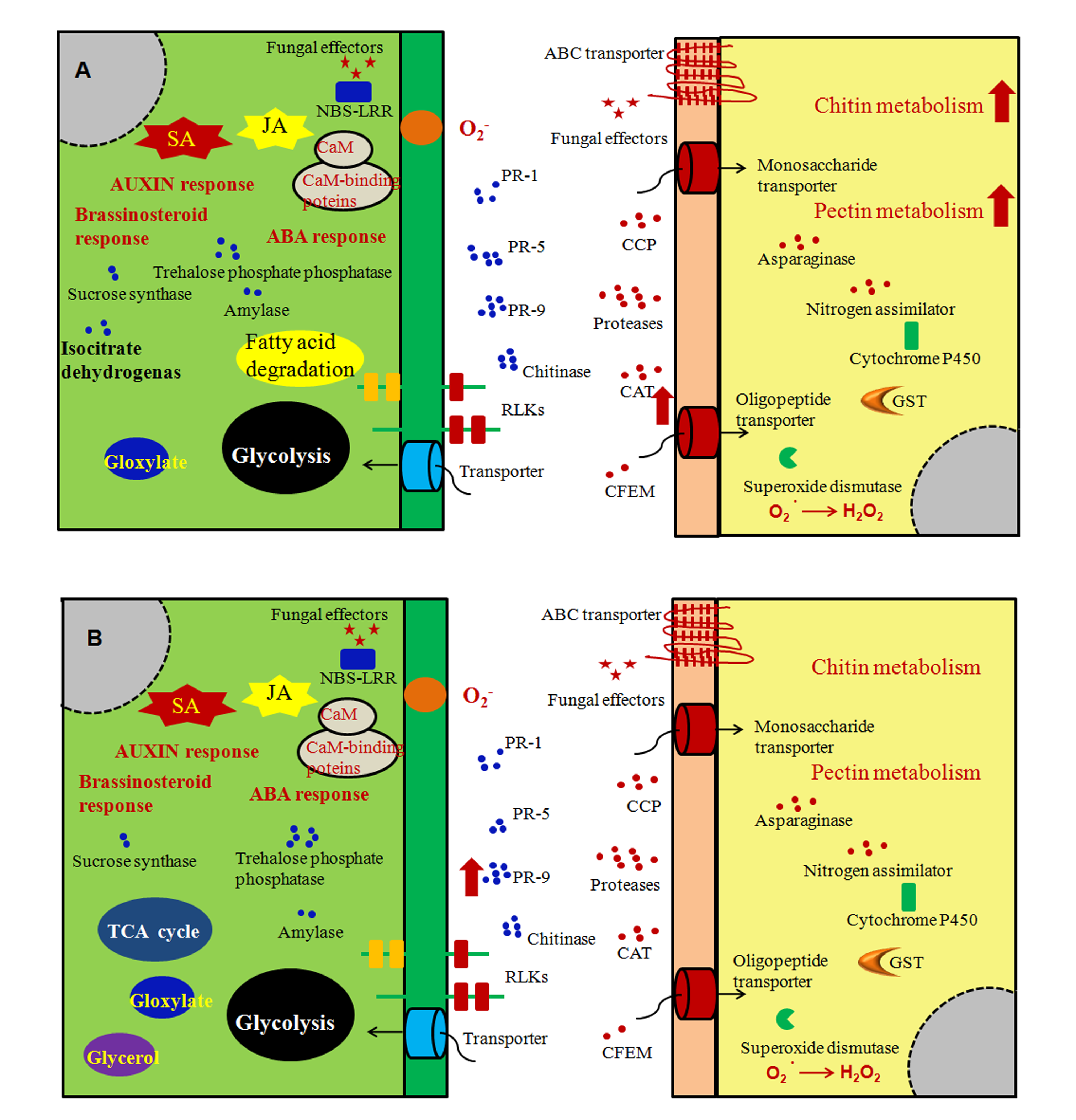

Supplement: Supplementary file 15 — Figure S8. A representative model of the interaction between different banana cultivars and Foc. (A) The response of BX to Foc TR4; (B) The response of NK to Foc TR4 CFEM: CFEM domain-containing proteins, CAT: Catalase, CCP: Cytochrome c peroxidase, GST: Glutathione S-transferase, ABC transporter: ATP binding cassette superfamily. (TIF 8095 kb) [file 12864_2019_5902_MOESM15_ESM.tif]

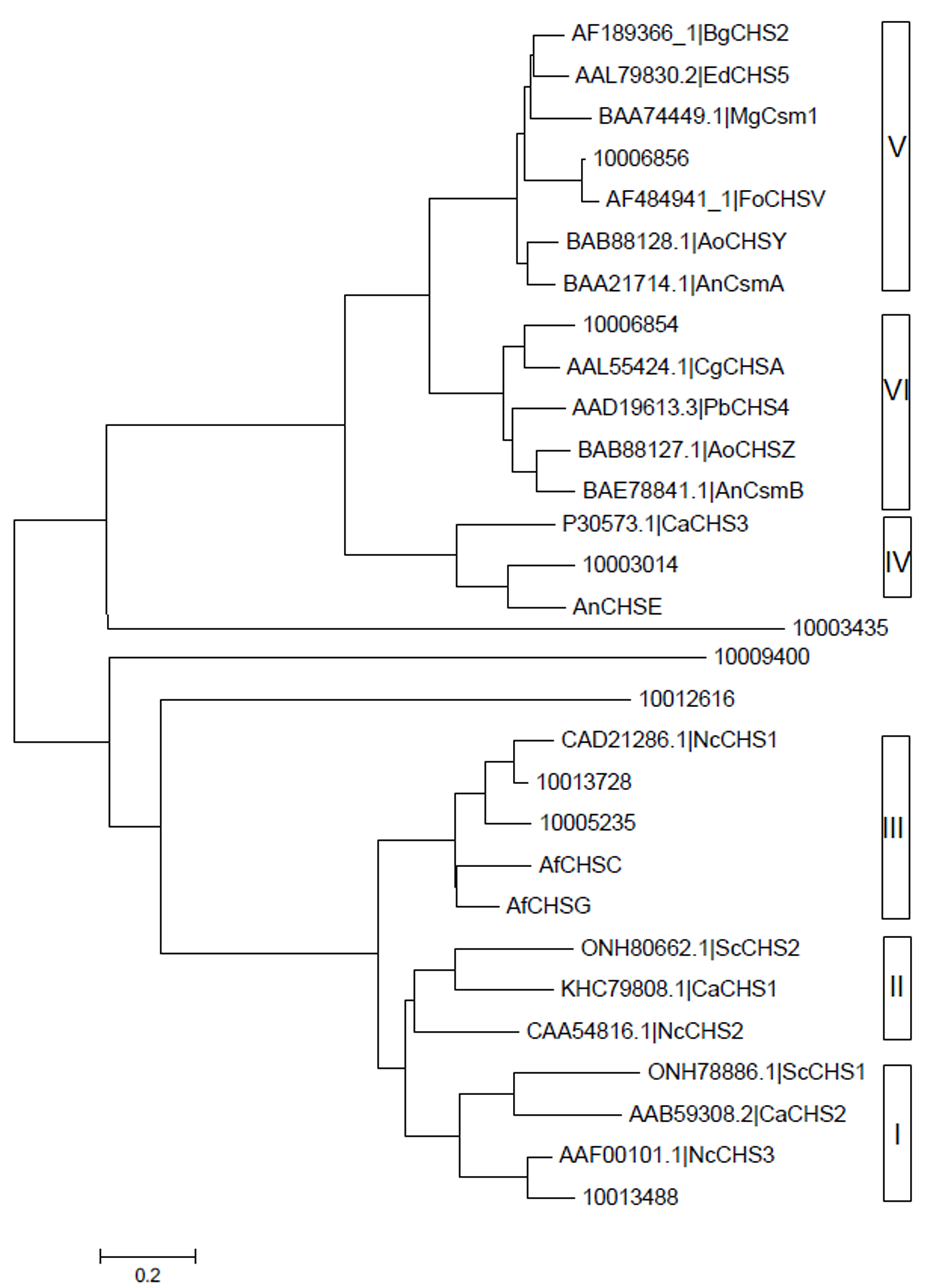

Supplement: Supplementary file 17 — Figure S9. Phylogenetic relationship of fungal chitin synthases. Sequences were taken from GenBank or genome projects. The neighbor-joining tree was constructed using clustal W program (http://clustalw.genome.jp/) and MEGA software version 5.0. The chitin synthase (CHS) are ScCHS1, 2 of Saccharomyces cerevisiae, NcCHS1, 2, and 3 of Neurospora crassa, CaCHS1, 2, and 3 of Candida albicans, BgCHS2 of Blumeria graminis, MgCsm1 of Magnaporthe grisea, FoCHSV of Fusarium oxysporum, CgCHSA of Colletotrichum graminicola, EdCHS5 of Exophiala dermatitidis, PbCHS4 of Paracoccidioides brasiliensis, AnCsmA of Aspergillus nidulans, AoCHSY and AoCHSZ of Aspergillus oryzae, and AnCsmB of Aspergillus nidulans. The sequence of AnCHSE,AfCHSC and AfCHSG were obtained from reference [58, 59]. (TIF 4315 kb) [file 12864_2019_5902_MOESM17_ESM.tif]
